# Recapitulating gestational progression: a high-throughput 3D bioprinted vascularized human placenta demonstrating stage-specific molecular and functional signatures of human pregnancy

**DOI:** 10.1038/s41598-026-61708-y

**Published:** 2026-07-28

**Authors:** Cristina Antich, Yu-Chi Chen, Justine Noel, Yanyan Qu, Yuhong Fang, Shayne Frebert, Dingyin Tao, Lauren S. Richardson, Ramkumar Menon, Min Jae Song, Marc Ferrer

**Affiliations:** 1 3D Tissue Bioprinting Laboratory, Division of Preclinical Innovation, National Center for Advancing Translational Sciences (NCATS), National Institutes of Health (NIH), Rockville, MD USA; 2 Analytical Chemistry Core, Division of Preclinical Innovation, National Center for Advancing Translational Sciences (NCATS), National Institutes of Health (NIH), Rockville, MD USA; 3 Division of Maternal-Fetal Medicine & Perinatal Research, Department of Obstetrics & Gynecology, The University of Texas Medical Branch, Galveston, TX USA

**Keywords:** Biological techniques, Cell biology, Developmental biology, Medical research, Physiology

## Abstract

The placenta is a highly dynamic organ that supports the growth and development of the fetus during gestation. Malfunction of the placenta causes disorders of the pregnancy, including preeclampsia and pre-term birth. The study of the pathophysiology of the placenta throughout pregnancy has been hindered by limited access to the human placenta and lack of predictive cellular and animal models. Here, we describe 3D bioprinted human placenta barrier (hPB) tissue models using primary human trophoblasts and stroma cells that recapitulate the physiology of the human placenta at early and late stages of gestation. These bioprinted vascularized hPB tissue models mimic the architecture and function early and late-stages human placenta, including barrier function, nutrient uptake, transporters activity and hormones secretion. Assembled in a 96-well transwell plate format, this deeply characterized platform provides a robust high-fidelity system for predictive screening of therapeutics and potentially hazardous agents for placenta diseases and safety, and a better understanding of placental pathophysiology.

**Supplementary Information:**

The online version contains supplementary material available at 10.1038/s41598-026-61708-y.

## Introduction

The human placenta is a dynamic organ that develops in the uterus during pregnancy and supports the growth and development of the fetus. It is fetal in origin and forms a barrier with maternal decidua basalis and serves as the functional interface between the mother and the developing fetus. The functions of the human placenta evolve during the different stages of the pregnancy, and include facilitating the transport of nutrients between the mother and the fetus, providing an immune barrier, protecting against xenobiotics, and secreting essential hormones required for pregnancy maintenance and termination^1–3^. Research on the physiology and pathology of the human placenta is limited by the ability to investigate during human pregnancy because of restricted accessibility, ethical considerations and lack of predictive in vitro and in vivo models. In vivo animal models have been invaluable, providing fundamental mechanistic insights into placental development; however, significant interspecies differences in placental anatomy and function can limit the direct translational relevance of these studies to human pregnancy^4–6^. Current non-animal approaches include ex vivo placenta tissues in which multiple relevant cell types and structural organization is preserved from the living organisms^7,8^, but have limited viability after being removed from the body and are challenging to obtain for the first and second trimester of pregnancy^9^. In vitro models offer simpler and more flexible tools to study various aspects of the placenta physiology. While immortalized cell lines have been foundational to the field, they often do not fully recapitulate the physiology and complexity of the native tissue^10–12^. Recently, three-dimensional (3D) models, including trophoblast spheroids and organoids from stem cells, have emerged as powerful tools for studying early-stage placental development^13–18^. Despite the improved throughput or physiological resemblance, the structural configuration of these models remains limited high-throughput measurements of key functional outcomes such as barrier integrity and transport. The compromise of this selective barrier is a hallmark of numerous placental pathologies, underscoring the critical need for models that can accurately recapitulate this physiological aspect. Recently, organ-on-chip platforms enabled achieving high tissue complexity demonstrating the placenta-chorion interface, containing seven different cell types from both the fetal and maternal side, interconnected through microchannel^19–24^. While the complex engineering platform allows for resembling the high order of dynamics in the interface, the complexity requires multiple, intricate manufacturing processes, and specialized equipment and expertise, hindering their adoption for high-throughput drug screening. An alternative approach involves the use of culture systems based on a multi-well transwell format, which provides an optimal platform to achieve the level of throughput and physiological relevance. Despite its static feature, the distinct apical and basal compartments recapitulate the basic physiology of maternal–fetal interface, enabling the formation of polarized cell layers. This configuration is particularly advantageous for efficient, high-throughput analysis of a wide range of placental functions. Furthermore, bioprinting enables the reproducible and scalable construction of 3D in vitro models by combining the automation needed for high throughput biofabrication with the spatial precision required to engineer vascularized tissue architectures.

Despite considerable progress in the advancement of in vitro models of the human placenta, challenges remain in the ability to reproduce the dynamic structural and functional changes that the placenta undergoes during pregnancy, including mimicking the environmental conditions that placenta is exposed at the different stages of gestation or the changes in the vascularization on the fetal side^25–29^. Most of the current in vitro human placenta models use choriocarcinoma cells (e.g., BeWo, JEG-3, JAR), which are valuable for standardized assays, but do not exhibit all the physiological features of the native tissue^30–33^. Recent placenta organoids and barrier models have been developed using TSCs^16^, which produce polarized placenta tissue models but did not recreate the different architecture and physiology of the placenta at different stages of gestation, did not include placenta stroma.

Therefore, here we describe the biofabrication of biomimetics tissue models of the human placenta barrier (hPB) that recapitulate architecture and function at various gestational stages of the pregnancy, using 3D bioprinting technology. These biofabricated hPB models were assembled using primary or immortalized trophoblasts cells obtained from term placentas and incorporated a vascularized stroma layer. To replicate key morphological and cellular elements of the hPB, trophoblasts (Tbs) and stroma cells (endothelial cells, fibroblasts and pericytes) were bioprinted on opposite sides of the membrane in the inserts of transwell plates. To recreate an early-stage placenta model, the hPB microtissues composed of a trophoblast bilayer and placental stroma vasculature were exposed to hypoxic conditions mimicking the uterine milieu. Conversely, for the late-stage placenta model, the hPB microtissues composed of a pseudostratified trophoblast layer and placental stroma vasculature were exposed to normoxia conditions of the maternal vasculature. The hPB models were validated using immunostaining, transcriptomics, proteomics and relevant functional readouts, including barrier function, nutrient uptake, transporter activity and hormone secretion. The biofabricated hPBs models were assembled in 96-well transwell plates, and we developed analytical methods that enable the measurement of placental functions including barrier activity, drug efflux, nutrients transport or secretion of relevant placental hormones, in rapid, cost-efficient assays amenable to high throughput screening.

## Results

### Bioprinted models of the vascularized human placenta mimicking early and late stages of gestation

**Fig. 1:**
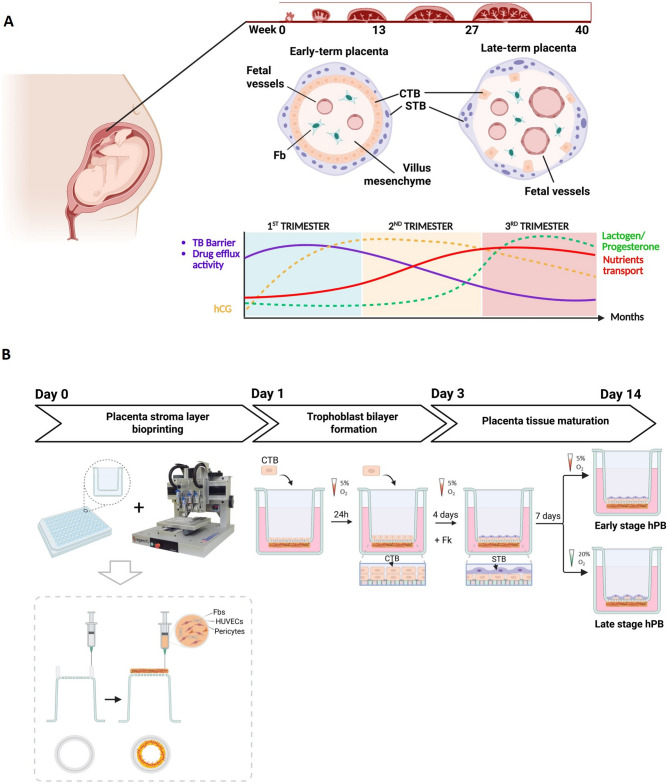
Schematic for the biofabrication of vascularized human placenta barrier (hPB) models at different stages of gestation. (**A**) Diagram of structural and functional changes of the placenta over gestational period. During the first trimester of gestation, chorionic villi of the placenta consist of a thick trophoblastic bilayer barrier composed of cytotrophoblasts (CTBs) and syncytiotrophoblasts (STBs), and underneath a mesenchyme containing fetal capillaries. As pregnancy progresses, the placenta surface expands and the CTBs become significantly less dense and the STB layer becomes predominantly mono-layered and thinner at term, with a denser fetal vasculature underneath, ideal for nutrient transport. In terms of functionality, the placenta in its early stages serves as a more selective barrier, while nutrient transport and hormone secretion activity increase over gestation. (**B**) Schematic of the workflow to generate the human placenta models (hPB) at different stages of gestation. The placental in vitro micro-tissues reproducing first and term stages of gestation were modelled by co-culturing trophoblasts and placental stroma cells (endothelial cells, fibroblasts and pericytes) in a 96-transwell platform. To develop the placenta stroma, a GelMA O-ring and stroma cells embedded in fibrin-based hydrogel (3 mg/ml Fibrinogen and 6% w/v Porcine Gelatin in PBS) were bioprinted on the opposing side of the transwell inserts. Stroma cells included Human Umbilical Vein Endothelial Cells (HUVECs), fibroblasts (Fbs), and Pericytes. Subsequently, the trophoblast layer was created by seeding a bilayer of cytotrophoblasts (CTB) on the apical side of the transwell insert. The CTB-STB bilayer was formed by differentiation for 4 days under Foskolin treatment and hypoxic conditions (5% O2). This early placental stage of growth was modeled by exposing the micro-tissue for 7 days in hypoxic conditions (5% O2) while late placental stage was modeled by exposing the micro-tissue for 7 days to normoxic conditions (20% O2), under differentiation basal media. Image created with BioRender.com.

The human placenta is a dynamic organ with extensive cellular plasticity and morphological and functional changes along the nine months of pregnancy. Architecturally, the placenta is a multi-layered organ including villous cytotrophoblasts (CTB), syncytiotrophoblasts (STB), and underlying stroma cells, which form a network of fetal vessels^2,34–37^. (Fig. 1A). At early-stages, the human placenta is subjected to low oxygen conditions (3–8%) of the uterine milieu and morphologically is characterized by a thick layer of trophoblastic cells with an underlying villus mesenchyme that contains fetal capillaries. During the later stages, it is subjected to normal oxygen levels (21%) of the maternal vasculature and consists of a thin, fused trophoblast layer (syncytium) and dense fetal vasculature. Functionally, during the early stages, the human placenta serves as a selective barrier, exhibiting high efflux activity. In contrast, throughout the later stages, it is primarily responsible for nutrients transport and hormone secretion, including progesterone, estrogens and lactogen (Fig. 1A)^36–38^. We aimed to assemble physiologically relevant human placenta models that faithfully mimic the morphology and function of placenta at the early and late gestational stages of pregnancy, and in a platform that enabled functional assays in high throughput screening format. Our bioengineered vascularized human placenta barrier (hPB) models were assembled using either primary trophoblastic cells from uncomplicated term pregnancies or immortalized human trophoblasts from uncomplicated term pregnancies and primary placental stroma cells (HUVECs, fibroblasts, and pericytes) also from uncomplicated term pregnancies. The process for the assembly of the vascularized hPB models described here is shown in the schematic diagram in Fig. 1B. Briefly, we first bioprinted a bioink that included a mixture of HUVECs, fibroblasts, and pericytes 7:1:0.7 (million cells/ml) in a fibrin-based hydrogel in a circular shape, on the basal side of a 96-well transwell insert, with a 1 mm PET membrane, and a GelMA O-ring to provide support to the bioprinted structure^39,40^. After 1 day incubation, a suspension of primary CTBs or immortalized trophoblasts (270 K cells/cm^2^) were added to the apical side of the membrane in the transwell insert to create a CTB monolayer. The following day, the process was repeated to create a second layer of CTBs. On day 4, forskolin was added to the apical media to induce differentiation of CTBs to STBs for 4 days. Tissues were incubated for additional 7 days in hypoxia conditions for the early-stage or 7 days in normoxia for the late-stage hPB models.

**Fig. 2:**
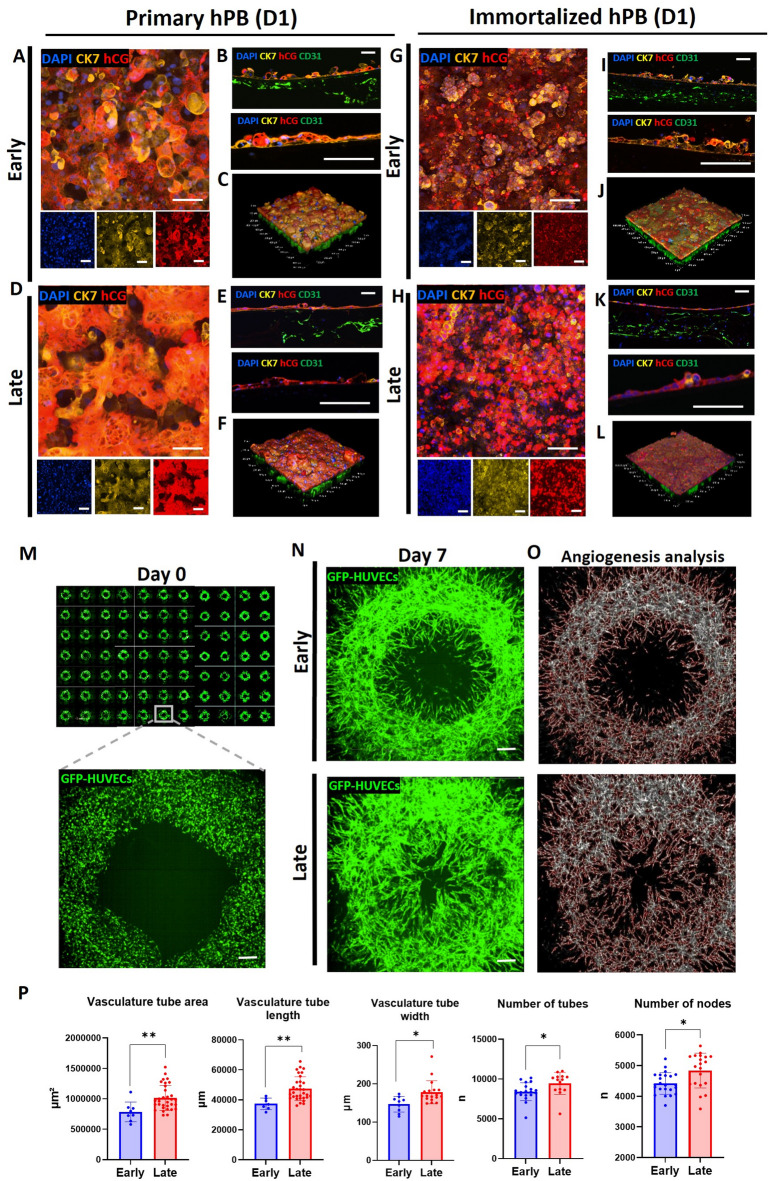
Structural analysis of human placenta 3D models at different stages of gestation. (**A**, **D**, **G**, **H**) Immunofluorescence images from confocal microscopy of the trophoblast bilayer from the human placenta models using trophoblast marker (CK7), syncytiotrophoblast marker (hCG) and nuclear marker (DAPI). (**B**, **E**, **I**, **K**) Histological sections of the human placenta models stained with specific markers for trophoblast layer (CK7, hCG) and stroma layer (CD31). (**C**, **F**, **J**, **L**) 3D view of the human placenta models. M) Images from confocal microscopy of the bioprinted placental stroma on the basal side of the transwell inserts at day 0. N) Images from confocal microscopy of the vasculature formation in the placental stroma at early and late gestational stage models after 7 days in culture. O) Images from the analysis of the vasculature in placenta stroma using SimA’s skeletonization tool for quantitative tube morphology assays. P) Quantification of the parameters related to vasculature and angiogenesis analyzed by SimA’s skeletonization tool in the placenta stroma at different gestational stage models. Cytokeratin 7 (CK7); Chorionic Gonadotropin hormone (CGH); Cluster of Differentiation 31 (CD31); 4′,6-diamidino-2-phenylindole (DAPI); Green Fluorescent Protein-expressing Human Umbilical Vein Endothelial Cells (GFP-HUVECs). Scale bar: 100 μm.

We first characterized the morphology of the two hPB models by using immunostaining and fluorescence confocal microscopy of the full tissues and histological cross-sections (Fig. 2 for donor 1 and Supplementary Fig. 1 for donor 2). Following seven days of culture under conditions that mimic early or late stages of gestation, hPB models exhibited physiologically relevant morphological differences: Using primary Tbs cells, the early-stage hPB model displayed a tissue with a thick multilayered trophoblast section (Fig. 2A-C for primary donor 1 and Supplementary Fig. 1A-C for primary donor 2), whereas in the late hPB model, the trophoblast layer appeared to be a multinucleated thin structure (Fig. 2D-F for primary donor 1 and Supplementary Fig. 1D-F primary donor 2), which is analogous to the structural organization of human placenta tissue in early and late-stages of pregnancy, respectively. The morphological differences were also appreciated in the hPB models produced using immortalized Tb cells, but to a lesser extent (Fig. 2G-L; Supplementary Fig. 1G-L). In all cases, the trophoblast layer in the early stage hPB models (both primary and immortalized cells) exhibited positive expression of the specific trophoblast lineage marker cytokeratin 7 (CK7), and STB expressed chorionic gonadotrophin hormone (hCG) in the outer layer, further demonstrating the appropriate trophoblast layering. In the late-stage models, the trophoblast layer formed with either primary or immortalized human placental cells exhibited higher expression of hCG in comparison to the early-stage trophoblast layer, indicating more matured STBs, which is a characteristic of advanced pregnancy stages. Histological cross-sections from both stages of either primary or immortalized vascularized hPBs confirmed the overall trophoblast layering and also showed the presence of placental stroma underneath the trophoblast bilayer(vasculature layer), as shown in green with CD31 immunostaining (Fig. 2B, E,I, K for primary donor 1; Supplementary Fig. 1B, E,I, K for primary donor 2).

The placental stroma cells in a fibrin-based hydrogel were bioprinted in ring shape to enable the quantitation of both vasculogenesis and angiogenesis using the fluorescence signal from GFP expressing HUVEC cells^39,40^(Fig. 2M). We observed formation of a dense network of vessels at day 7, for both early- and late-stage models in the bioprionted ring area (vasculogenesis) (Fig. 2N). The analysis of GFP fluorescence signal from the HUVECs cells using high-content imaging (Fig. 2O and Supplementary Fig. 2) revealed a higher level of angiogenesis in late- over early-stage hPB, as measured with an increased vessel area inside the ring structure, a greater number of nodes, an elevated total vessel tube length (47416 μm vs. 37356 μm, *p* = 0.0026, Student’s t-test, Fig. 2P), and a vessel diameter (178.1 μm vs. 147 μm, *p* = 0.0101, Student’s t-test, Fig. 2P). The increase in vessel area in the late-stage (1015638 μm² vs. 784477 μm², *p* = 0.0323, Student’s t-test) further demonstrates a late-stage placenta phenotype in the hPB under normoxia conditions.

### Transcriptomic study of the vascularized hPB models of early and late stages of gestation

**Fig. 3:**
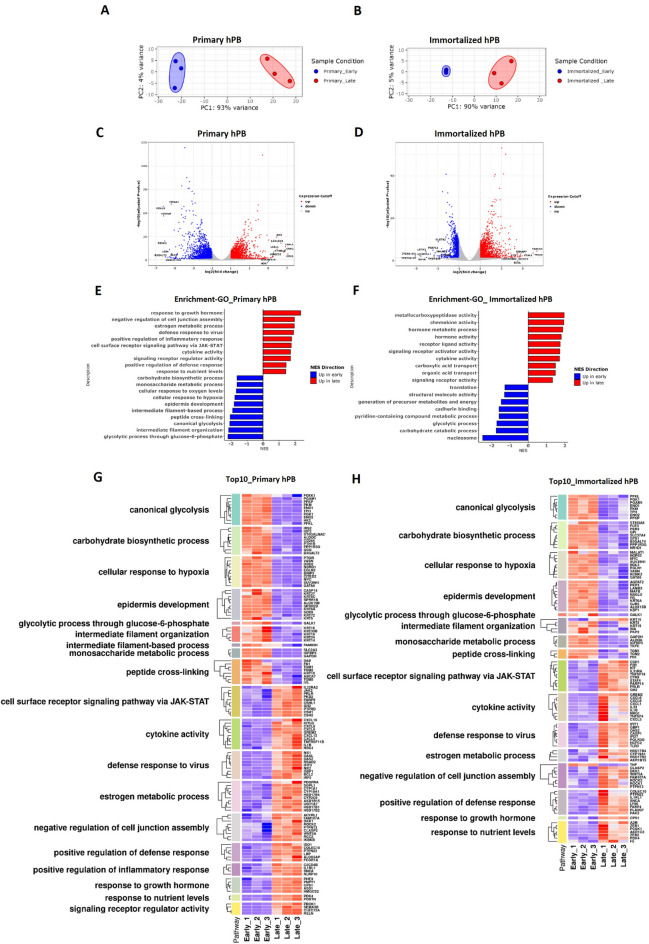
Transcriptomic profiling of human placenta 3D models at different stages of gestation. (**A**-**B**) PCA separation of placenta models using the differentially expressed genes (DEG). (**C**-**D**) Volcano plot of DEGs from placenta models, with genes higher in the early-stage in blue, and those higher in the late-stage in red. (**E**-**F**) Box plots of biological processes that were differentially expressed between gestational stages of placenta model. (**G**-**H**) Heatmap of top DEGs in the early-stage versus late-stage placenta samples.

We further validated the vascularized hPB models at early and late gestational stages by transcriptomics analysis with RNA sequencing. Gene transcripts expression levels were compared for early- and late-stage hPB models assembled from primary Tb cells (donor 1) or immortalized cells (donor 1) (Supplementary Table 1 for hPB from primary Tb; and Supplementary Table 2 for hPB from immortalized Tbs). Gene expression signatures obtained from N = 3 technical replicates of each of the four hPB models were analyzed by principal component analysis (PCA) which was able to group the samples according to gestational age and cell type (Figs. 3A and 8B). The differentially expressed genes (DEG) analysis of hPB at early and late stages derived from primary (Supplementary Table 3) and immortalized Tbs cells (Supplementary Table 4) are shown in volcano plots and revealed 1,894 upregulated and 1,913 downregulated genes in hPB samples obtained from the primary Tbs at the late-stage, compared to the early-stage model (adjusted p-value (FDR) threshold of 0.05 and a log2 fold change (log2FC) of 1) (Fig. 3C). Gene Ontology enrichment analysis revealed that in the hPB models assembled with primary or immortalized trophoblasts, genes related to biological processes such as carbohydrate metabolism and glycolysis, response to oxygen levels, and structure organization, were upregulated in the early stage hPB. Conversely, genes involved in hormone activities, cell signaling activation and regulation, transport and inflammatory responses upregulated in the later-stage model (Fig. 3E and F). The ten most differentially expressed genes for each relevant biological process expressed in the different hPB models are shown in Fig. 3G and H, respectively. The complete list of all differentially expressed genes in hPB samples derived from primary and immortalized Tbs is in Supplementary Tables 5 and 6, respectively. The enrichment score (ES) plots for all differentially expressed gene sets are shown in Supplementary Fig. 3. We also identified clusters of highly differentially expressed genes associated with the terms “cell cycle,” “metabolism,” “response to xenobiotic stimulus”, “transport”, “hormone activity”, “Extracellular matrix” and “blood vessel development,” using Gene Ontology analysis (GO)^86–96^ [Supplementary Table 7 (Primary) and 8 (Immortalized), and Supplementary Note 1]

### Proteomic study of hPB models at early and late stages of gestation

**Fig. 4:**
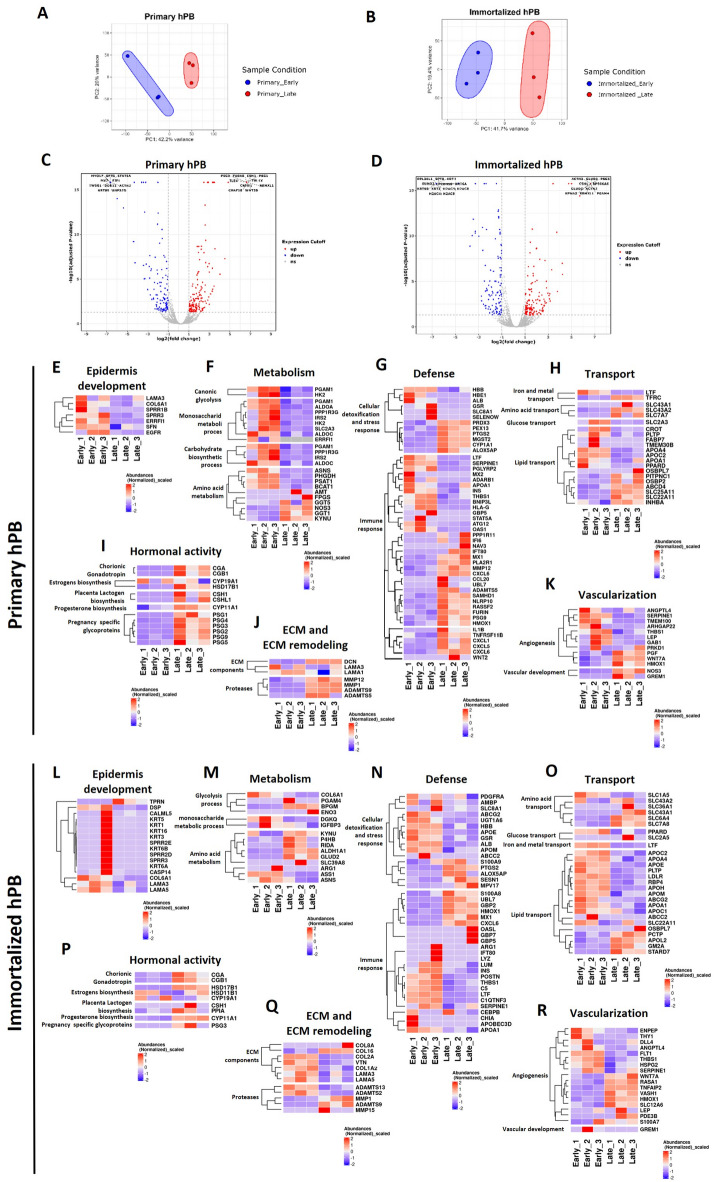
Proteomic profiling of human placenta 3D models at different stages of gestation. (**A**- **B**) PCA separation of placenta models using the differentially expressed proteins. (**C**-**D**) Volcano plot of differentially expressed proteins between human placenta models, with higher expression in the early-stage in blue, and those higher in the late-stage in red. (**E**-**R**) Heatmap of differentially expressed proteins involved in selected biological processes in early-stage versus late-stage placenta samples, using primary (**E**-**K**) and immortalized (**L**-**R**) Tbs.

We further validated the physiological relevance of the hPB models by assessing the differences in protein levels using liquid chromatography coupled to mass spectrometry proteomics analysis. The protein content of hPB tissues using same primary (donor 1) and immortalized (donor 1) Tbs as used for the transcriptomics analysis, at early and late gestational stages, was analyzed. PCA of the protein levels grouped hPB models from both Tbs by gestational age (Fig. 4A, B), showing a substantial correlation in protein expression patterns (complete list of protein levels is in Supplementary Table 9 for hPB from primary; and Supplementary Table 10 for hPB from immortalized). The differential protein expression analysis identified fewer significant proteins compared to transcriptomics (Supplementary Tables 11 and 12). The volcano plots reveal 382 upregulated and 195 downregulated proteins in hPB derived from the primary Tbs at the late-stage in comparison to the early-stage model (Fig. 4C). In the late-stage hPB derived from immortalized Tbs, 300 proteins were upregulated, and 332 proteins were downregulated relative to the early-stage model (Fig. 4D). Analysis of differentially expressed proteins between gestational stage models utilizing Gene Ontology enrichment analysis and GO keywords overlaps with the transcriptomics data, including epithelial barrier development, nutrient transport, defense, extracellular matrix, angiogenesis and hormonal activity (Figs. 4E-R, Supplementary Note 2). Overall, the comparative analysis of the log2 fold change of deferentially expressed genes at the transcript and protein levels demonstrated a strong correlation in gene clusters related to epidermis development, metabolism, hormone activity, extracellular matrix, and defense activity in placenta samples from primary and immortalized Tbs sources (Supplementary Figs. 4 and 5, respectively).

### Barrier integrity of the hPB models

**Fig. 5:**
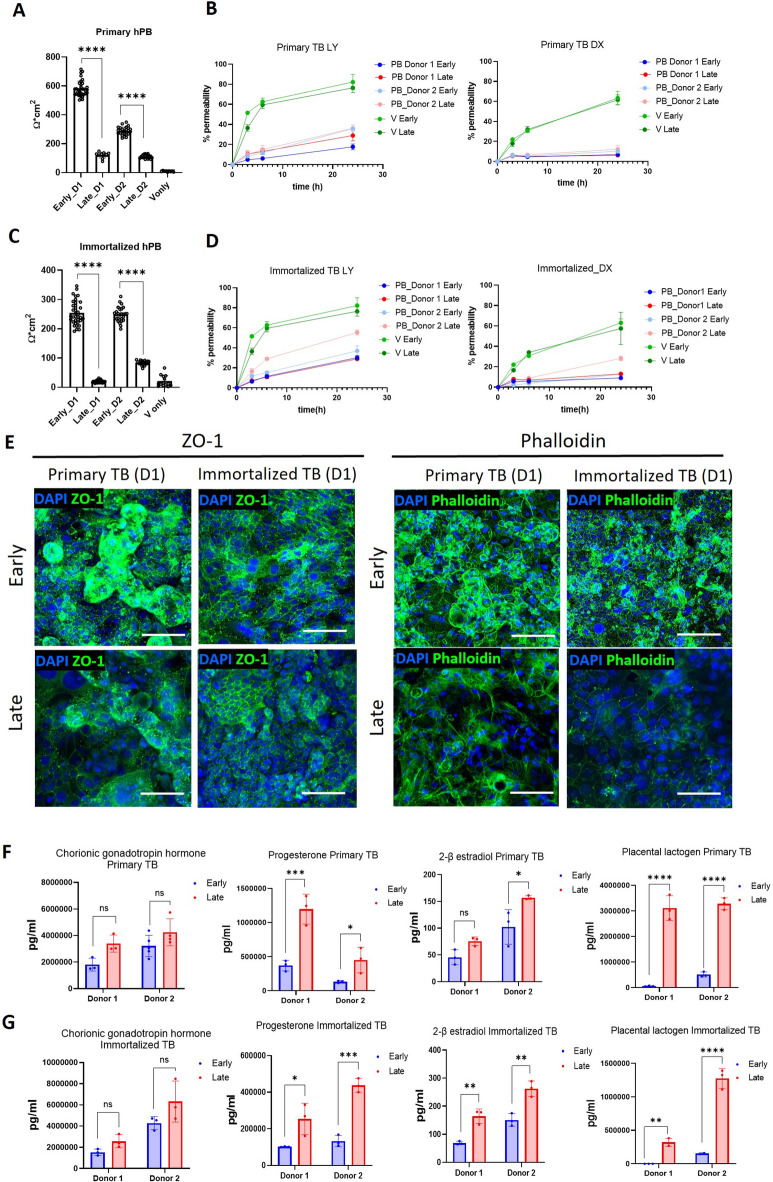
Barrier integrity and hormonal activity of human placenta 3D models changed at different stages of gestation. (**A** and **C**) TEER measurements from placenta models using different cell donors. (**B** and **D**) Permeability assay using fluorescent dyes with different molecular weight. Results from spectrophotometry revealed the capability to maintain resistance against different dyes permeation. LY: Lucifer Yellow; DX: Dextran; kDa: Kilodalton. PB: human placenta 3D model; V: Placenta stroma (**E**) Tight junction protein ZO-1 and F-actin immunofluorescent staining images of trophoblast layer in placenta models (donor 1) after maturation culture time. Scale bar: 100 μm. Quantification by enzyme-linked immunosorbent assay of four pregnancy hormones synthetized by the human placenta from primary Tbs (**F**) and immortalized Tbs (**G**): human chorionic gonadotropin, Progesterone, Estrogens and Placental lactogen. Supernatants were collected from the apical side of the hPB models on day 10 and analyzed using ELISA kits for each hormone. Each experiment was done in biological replicate (*N* = 3), error bars represent standard deviation. Statistical significance was determined by two-way ANOVA for TEER value and hormone level differences between placental stage models and Multiple unpaired t-test for permeability differences (**** *P* < 0.0001,*** *P* < 0.001, ** *P* < 0.01, * *P* < 0.05, ns *P* > 0.05).

To quantitatively assess the formation of the hPB barrier, we measured transepithelial electrical resistance (TEER) and paracellular permeability. TEER studies revealed that the trophoblast layer derived from both primary and immortalized cells induced a significant increase in transepithelial resistance, compared to the vascularized stroma layer alone (p = < 0.0001, two-way ANOVA), and TEER values were lower in late-stage values were lower in the late- vs. early-stage hPB models (579.3 Ohms/cm² vs. 121.3 Ohms/cm² for primary, and 288 Ohms/cm² vs. 110.2 Ohms/cm² for immortalized, p = < 0.0001, Student’s t-tests). Figure 5A and C (primary and immortalized trophoblasts cells, respectively) indicate that the incorporation of Tbs layer raised TEER values between 10 to 40-fold compared to placenta stroma alone: early-stage hPB from primary donors exhibited values of ≥ 300 Ohms/cm², while late-stage hPB exhibited values ≤ 120 Ohms/cm². Similar observations were noted in the hPB with immortalized Tbs, with resistance values ≥ 250 Ohms/cm at early-stage, while late-stage hPB models had lower values (≤ 90 Ohms/cm^2^).

We further tested hPB barrier integrity measuring permeability of two molecules of different molecular weights: 457 Da Lucifer Yellow (LY) and 10 kDa Dextran-TAMRA (DX). Leakage from the apical (lumen on the mother side) to the receiver compartment (fetal side) was monitored 3 h, 6 h and 24 h (Fig. 5B and D). The acellular membrane permeability was used to standardize the permeability in the tissue samples. As shown in Fig. 5B and D, the stroma layer exhibited significant permeability to LY, facilitating > 50% penetration of the acellular membrane into the fetal compartment after 3 and 6-hour incubation, and 72.5% permeation after 24 h. The permeation of DX was still very evident but to a lesser extent (21.5%, 30.33% and 59.7% of the membrane permeability after 3, 6 and 24 h, respectively). Conversely, hPB produced from primary and immortalized trophoblasts cells maintained a very low permeability of both molecules at all time points. Early stage of hPB from the primary Tbs exhibited permeability values < 7% and < 20% of acellular membrane permeation for LY after 6 and 24 h, respectively, and < 5% for DX. Late-stage hPB exhibited significantly more permeability, with values < 17% and < 34% for LY after 6 h and 24 h respectively (13.11% vs. 6.13%, *p* = 0.0215 for 6 h, 28.99% vs. 17.74%, *p* = 0.0004 for 24 h), and < 7% for DX (6.89% vs. 6.25% at 24 h, *p* = 0.0183, Student’s t-tests). The second donor of Tbs exhibited a similar tendency, but without significant differences between stages (p-value > 0.05, Student’s t-tests). Comparable results were observed in hPB assembled with immortalized Tbs, allowing only 11% permeation of LY and < 30% after 6 and 24 h, respectively. For DX, the permeability was < 7% and < 14% after 6 and 24 h, respectively. In this case, the permeability of second donor of Tbs was significantly higher in late stages (LY: 29.08% vs. 15.19% for 6 h, p-value < 0.000, and 55.27% vs. 36.94% for 24 h, p-value < 0.0001; DX: p-value < 0.001 for 6 h and 24 h, Student’s t-tests).

We also assessed the expression of zonula occludens-1 (ZO-1) in the trophoblast layer and the actin cytoskeleton using phalloidin labeling by immunostaining. Early-stage hPB models exhibited strong expression of ZO-1 and phalloidin, with distinct cell-to-cell boundaries throughout the surface, while the later stage model showed decreased density and reduced expression of both markers (Fig. 5E and Supplementary Fig. 6). This data supports the higher TEER values and lower permeability seen in the early stage hPB models, as indicative of a higher barrier integrity.

### Hormone secretion of the hPB models

A major activity of the placenta is the dynamic secretion of different hormones to sustain pregnancy at different stages. Chorionic gonadotropin hormone, progesterone, estrogens and placental lactogen are hormones secreted by the placenta and key biomarkers for different stages of gestation. Here, we analyzed the production of those hormones in our models at the end of the maturation time using ELISA assays (Fig. 5F and G for hPB using primary and immortalized, respectively). Chorionic gonadotropin hormone levels in hPB using either primary or immortalized Tbs had a trend towards increased production in late-stage models; however, these differences were not statistically significant. Progesterone levels secreted by hPB models demonstrated a correlation with gestational age, exhibiting a significant increase of ≥ 3.41-fold in the late-stage model with primary Tbs (1196 μg/ml vs. 370.45 μg/ml for donor 1 (*p* = 0.0003), 1325.59 μg/ml vs. 452.2 μg/ml for donor 2 (*p* = 0.04), Two-way ANOVA) and ≥ 2.5-fold for hPB models assembled with immortalized Tbs [253.927 μg/ml vs. 101.7 μg/ml for donor 1 (*p* = 0.0111), 437.279 μg/ml vs. 133.06 μg/ml for donor 2 (*p* < 0.0001), Two-way ANOVA). Quantitative measurements of estrogens, particularly 2-β estradiol, revealed markedly reduced levels compared to the other hormones measured in this analysis. However, we still detected significantly higher amounts of 2-β estradiol in late-stage hPB models derived from primary Tb donor 2 (102.42 vs. 157.12 pg/ml, *p* = 0.0125, Two-way ANOVA) and both donors of immortalized Tbs [69 vs. 164.31 pg/ml, for donor 1 (*p* = 0.0047), and 150.9 vs. 262.4 pg/ml for donor 2 (*p* = 0.0017), Two-way ANOVA]. We observed an increase in production of placental lactogen from early-stage hPB models derived from primary Tbs to late-stage models. A significant increase in production of placental lactogen was also observed in late-stage models derived from primary [3107.39 vs. 608.37 μg/ml, for donor 1 (*p* < 0.0001), and 3279.31 vs. 516.42 μg/ml for donor 2 (*p* < 0.0001), Two-way ANOVA] and immortalized [321 vs. 0 ug/ml, for donor 1 (*p* = 0.0022), and 1276.52 vs. 150 μg/ml for donor 2 (*p* < 0.0001), Two-way ANOVA] hPB, This data indicates that our early and late-stage models, using primary or immortalized Tbs, effectively recreated the hormonal alterations throughout gestation found in the human placenta.

### Nutrients transport activity of the hPB models

**Fig. 6:**
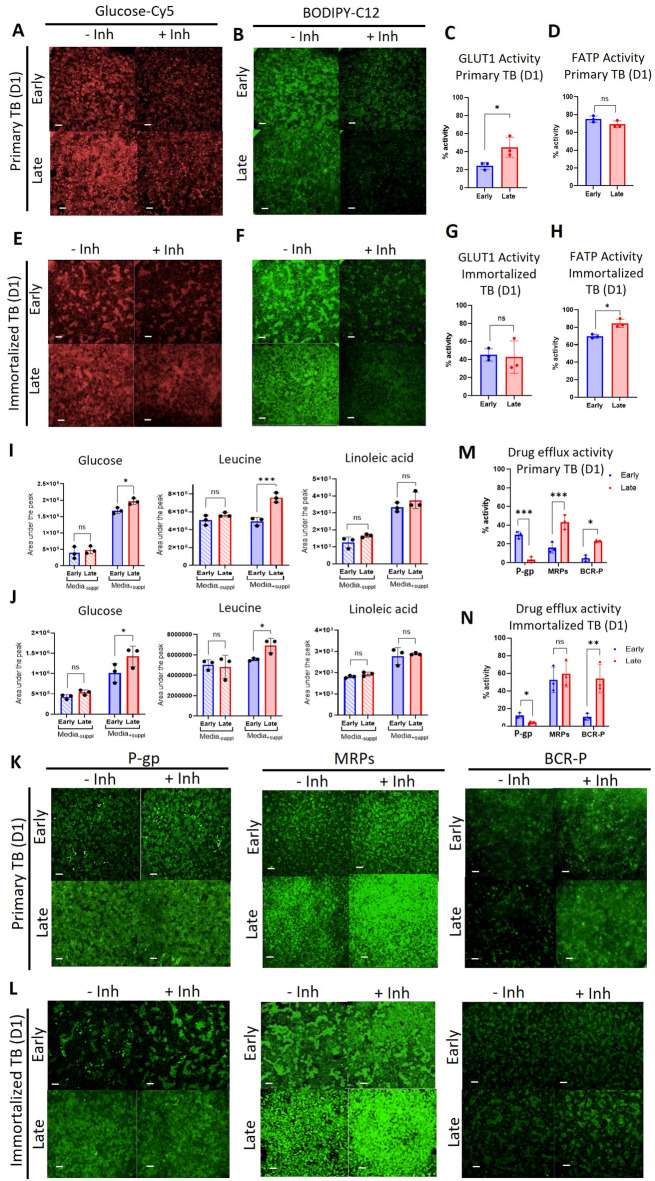
Nutrients transport and drug efflux activity of human placenta 3D models changed at different stages of gestation. (**A** and **E**) Confocal fluorescence imaging of Glucose-Cy5 uptakeby the trophoblasts layer of the human placenta models at different gestational stages. (**C** and **G**) Nutrient uptake activity by main transporter of glucose (GLUT1). (**B** and **F**) Confocal fluorescence imaging of C12-BODIPY uptake by the trophoblasts layer of the human placenta models at different gestational stages. (**D** and **H**) Nutrient uptake activity by main fatty acid transporter (FATP2). (**I** and **J**) Analysis of nutrient transfer activity of placenta models at different gestational stages by targeted metabolic profile of representatives of carbohydrates, proteins, and lipids (glucose, leucine, and linoleic acid, respectively). (**K**, **L**) Confocal images of trophoblasts from human placenta 3D models at different gestational stages containing fluorescent substrate compounds of P-gp, MRPs and BCR-P pumps (green), after incubation with and without specific inhibitors for P-gp, MRPs and BCR-P pumps. (**M**, **N**) Transporter activity of P-gp, MRPs and BCR-P pumps in trophoblasts from human placenta 3D models at different gestational stages. Scale bar:200 μm. Each experiment was done in biological replicate (*N* = 3), error bars represent standard deviation. Statistical significance was determined by two-way ANOVA for nutrient uptake and transfer values, and efflux activity differences between placental stage models and Multiple unpaired t-test for nutrient uptake activity differences between placental stage models. (**** *P* < 0.0001,*** *P* < 0.001, ** *P* < 0.01, * *P* < 0.05, ns *P* > 0.05).

Nutrient transport from the maternal to the fetal side is a critical function of the placenta to ensure the development and growth of the fetus during pregnancy. To evaluate the apical-to-basal transport of nutrients through the vascularized hPB barrier models, we performed two distinct sets of experiments: one to quantify the initial cellular uptake of nutrients by the trophoblast layer, and another to measure the net directional transport of nutrients across the placental barrier from the apical to the basal compartment. First, to evaluate cellular uptake, we analyzed the absorption of glucose and fatty acids labeled with distinct fluorophores (Glucose-Cy5 for glucose and C12-BODIPY for fatty acids) by the trophoblast layer of the hPB at different gestational stages, for 1 h exposure. Confocal fluorescence imaging of Glucose-Cy5 and C12-BODIPY uptake by hPB assembled with primary and immortalized trophoblast cells revealed that the absorption rate levels are influenced by the developmental stage, with a general trend of increased uptake in the late-stage hPB models. For Glucose-Cy5, primary and immortalized Tbs from late-stages shown significant higher fluorescence values [1977 vs. 1438 RFU for primary (*p* = 0.0023), 3948 vs. 2406 RFU for immortalized (*p* = 0.0019), two-way ANOVA, Fig. 6A, E and Supplementary Fig. 8 A, C]. A similar pattern was observed when we examined the second primary trophoblast donor, showing noticeably more uptake in the late-stage model (2488 vs. 1505 RFU, *p* < 0.0001, two-way ANOVA), but no differences were observed in hPB models with immortalized Tbs (Supplementary Fig. 7 A, E and Supplementary Fig. 8B, D). To further validate that the uptake of Glucose-Cy5 was via glucose transporters (GLUTs), we tested glucose uptake in the presence of glucose transporter inhibitors. Several selective inhibitors with different selectivity for the main glucose transporter in the placenta were tested and GLUT1-IN-1 was the only one that significantly diminished glucose absorption for all tissues (Supplementary Fig. 9). Glucose uptake data measured by fluorescence imaging demonstrates that treatment with GLUT1-IN-1 significantly reduced the cellular uptake of Glucose-Cy5 in hPB assembled with primary trophoblasts, at both stages of gestation [1438 vs. 1088 RFU for early (*p* = 0.025), 1977 vs. 1092 RFU for late (*p* < 0.0001), two-way ANOVA]. The reduction was considerably greater in the late-stage models derived from donor 1, indicating elevated GLUT1 activity (45.11 vs. 24.36%, *p* = 0.034, two-way ANOVA, Fig. 6C). The second donor exhibited less reduction of glucose uptake in late-stages compared to early-stages (Supplementary Fig. 7 C). A similar trend was noted in hPB assembled with immortalized trophoblasts (donor 1), showing increased fluorescence values of Glucose-Cy5 in late-stage model prior to inhibitor treatment (3948 vs. 2406 RFU, *p* = 0.0019, two-way ANOVA); however, in this case, the GLUT1 activity remained similar across the hPB of different stages from both donors (Fig. 6D and Supplementary Fig. 7D).

The evaluation of fatty acid (FA) uptake by the hPB using fluorescence imaging of C12-BODIPY did not demonstrate clear trends between the two gestational hPB models (Fig. 6B, F and Supplementary 7B, F). In those derived with primary trophoblasts, significantly greater fluorescence levels were found exclusively in the late-stage from donor 2 only (6928 VS 5037 RFU, *p* = 0.0006, two-way ANOVA, Supplementary Fig. 8E, F). We tested several FA transporter inhibitors (Supplementary Fig. 10) and found that Lipofermata was the most potent inhibitor of FA uptake in both cell types, regardless of the stage. The subsequent studies assessing the effects of inhibitors of fatty acid transporters demonstrated elevated transporter activity across all conditions, evidenced by a marked reduction in fluorescence intensity of BODIPY-C12 post-treatment with Lipofermata (*p* < 0.0001, two-way ANOVA), although no significant differences in activity were noted among the different hPB models derived from primary trophoblasts (Fig. 6D and Supplementary Fig. 7D). hPB models derived from immortalized trophoblasts exhibited divergent responses (Supplementary Fig. 8G, H). However, significantly higher activity of fatty acid transporters was observed in the more advanced stage of hPB with cells from either donor [84.55% vs. 69.69% for donor 1 (*p* = 0.0146), 69.46% vs. 59.49% (*p* = 0.0097), two-way ANOVA, Fig. 6H, and Supplementary Fig. 7H).

To further investigate the directional transport of nutrients across the hPB models to the fetal side, we conducted targeted metabolomics analysis of several metabolites in the medium on the basal (fetal) side of the transwell inserts. The metabolites studied included glucose, leucine, and linoleic acid, which were chosen as representatives of carbohydrates, proteins, and lipids, respectively (Supplementary Note 3). Figure 6I, J demonstrates that the addition of metabolites into the media of the apical chamber significantly enhanced the directional transport of these nutrients to the fetus, especially glucose and leucine, in the late-stage model derived from primary [Glucose: 1964076 vs. 1680876 AUC (*p* = 0.0495), leucine: 7583812 vs. 3218626 AUC (*p* < 0.0001), two-way ANOVA] and immortalized Tbs [Glucose: 1421871 vs. 1014830 AUC (*p* = 0.0434), leucine: 6917890 vs. 5017982 AUC (*p* = 0.0426), two-way ANOVA].

### Drug efflux activity of the hPB models

Throughout pregnancy, drug transporters in the placenta protect the fetus from various endogenous and exogenous xenobiotics that could harm the fetus by actively transporting them back to the maternal side. We investigated the functionality of major drug transporters, including P-glycoprotein (P-gp), the multi-drug resistance protein (MRP) family, and Breast Cancer Resistance Protein (BCR-P), in the hPB at different gestation stages by evaluating the efflux of fluorescent substrates and blockers specific to each transporter. We used Rhodamine 123 (Rho123) as a fluorescent dye that is a P-gp substrate^42^, and Cyclosporine A (CSA), as a P-gp inhibitor^41^. We examined the function of MRPs utilizing the cell tracker 5-chloromethylfluorescein diacetate (CMFDA) as a specific substrate and MK571 as an inhibitor of the MRP family^42,43^. And the activity of BCR-P was assessed utilizing BODIPY-Prazosin as the substrate and ko-143 as the inhibitor of this pump^44,45^. The concentration of blockers used was based on a preliminary assessment, as shown in Supplementary Fig. 11. In the early-stage hPB model, with either primary or immortalized Tbs, there was a significant increase in the uptake of Rho123 with CSA treatment for the primary Tbs [Donor 1: 637.9 vs. 438 RFU (*p* = 0.0006), Donor 2: 489.7 vs. 336.4 RFU (*p* = 0.0434), two-way ANOVA, Supplementary Fig. 7I, J], and for the immortalized Tbs [Donor 1: 530.7 vs. 448.9 RFU (*p* = 0.0035), Donor 2: 1532 vs. 1415 RFU (*p* = 0.0053), two-way ANOVA, Supplementary Fig. 8I-L), indicating P-gp activity. However, for the late-stage hPB model, we did not detect any changes in Rho123 uptake upon treatment with CSA suggesting no substantial P-gp activity (Fig. 6K, L, Supplementary Fig. 7I, J and Supplementary Fig. 8I-L). Treatment with the MRP inhibitor MK571 led to an increased accumulation of CMFDA in hPB with primary Tbs for early-stage and for late-stage; and also for the hPB model with immortalized Tbs for early-stage and for late-stage, suggesting enhanced MRP transporter activity in the late-stage placental models (Fig. 6K, L, Supplementary Fig. 7I, J and Supplementary Fig. 8M-P). Treatment with the BCR-P inhibitor ko-143 revealed that the BCR-P activity as measured by BODIPY-Prazosin uptake was dependent on cell type (Fig. 6K, L, Supplementary Fig. 7I, J and Supplementary Fig. 8Q-T). In summary, these results demonstrate that the activity of the different transporters changes from early to late-stage in the different hPBs models. (Fig. 6M, N, Supplementary Fig. 7 K, L).

## Discussion

The development of placental models that that incorporate physiologically relevant cells, recapitulate the organ’s dynamic physiology at different stages of pregnancy, and are amenable to functional testing in a robust, reproducible, high-throughput format remains a critical objective in reproductive biology and toxicology. While many models of the placenta have been previously described, most do not meet all these requirements simultaneously, as each system possesses unique, context-dependent strengths and inherent limitations. In vivo animal models, despite species-specific anatomical differences that limit direct translation to human pregnancy, have historically provided indispensable mechanistic insights into placental development. Traditional 2D culture of immortalized cell lines, while lacking the structural and physiological complexity of native tissue, remain highly valuable for standardized, high-throughput assays. Furthermore, advanced in vitro systems, including trophoblast stem cells (TSCs) and pluripotent stem cell-derived models, have significantly improved our ability to study early-stage developmental dynamics. The bioprinted 3D hPB model described in this study represent a significant step toward bridging the gap between biological complexity and scalable analysis. Our versatile biofabrication approach integrates key native architectural and functional features of the human placenta at different gestational stages, enabling models of targeted physiological relevance within a high-throughput screening format.

Our biofabricated hPB models shows: 1) early- and late-stage placenta architecture achieved using layering protocols in conjunction with hypoxia and normoxia conditions to mimic first trimester uterine milieu environment and later trimesters maternal blood environment, respectively. During the initial stages of pregnancy, the placenta villi are covered by both cytotrophoblasts and syncytiotrophoblasts on the villus surface, resulting in the formation of a bilayered trophoblastic barrier. As pregnancy progresses, the placenta expands and the cytotrophoblasts become significantly less dense. Consequently, the syncytiotrophoblast layer becomes predominantly mono-layered and thinner at term, while preserving its structural integrity^35–37^. Our immunostaining studies of full-thickness tissue and histological cross-sections corroborated these morphological features across stages, using both primary and immortalized Tbs. The stroma layer beneath the trophoblastic barrier in the bioprinted hPB displayed denser and more vascularized structure in the late-stage model, consistent with observations throughout gestation progression^28,46^. 2) large increases in pregnancy hormones secretion in the late-stage, especially progesterone and placenta lactogen. The hormones synthesized by trophoblast cells comprise hCG, sex hormones (progesterone, estrogens) and placental lactogen (hPL), whose amounts and gestational profiles in maternal blood fluctuate throughout time. hCG is believed to be the initial placental hormone that influences the mother^47^, promoting the preservation of luteal function, cytotrophoblast development, maternal immunotolerance, and endometrial angiogenesis. During pregnancy, hCG levels demonstrate a fast increase, peaking near the conclusion of the first trimester. The levels subsequently decreased to ≈ 30% of the peak value and sustained a high level for the remainder of the pregnancy^48^. Instead, the remaining hormones become predominant later in pregnancy, with a significant increase in their levels in the second trimester and continuing until the end of the gestational period^38^. Progesterone and estrogen are essential for the maintenance of pregnancy, as they are involved in maintenance of fetal membrane integrity, myometrial quiescence, cervical closure, placental neovascularization, immunotolerance, and the mammary gland development^49,50^. hPL regulates maternal metabolism to fulfill the energy demands of the developing fetus^51^. Our results from transcriptomics and proteomics revealed a notable elevation of chorionic gonadotrophin and placental lactogen hormones, along with proteins linked to the biosynthesis of estrogens and progesterone in late-stage models produced from both cell lines. The quantification of hormones via ELISA techniques corroborated these findings, indicating that progesterone, estrogens, and placental lactogen levels from both Tb types displayed a gestational stage dependence, with significantly elevated levels in the late-stage models of 3D hPB. Conversely, comparable quantities of hCG were detected between stages in models derived from either Tbs. According to the previous studies reporting the levels of expression of those hormones over gestation^51–54^, our data suggests that early and late hPB models recapitulated first phases of first trimester and third trimester of gestation, respectively. And 3**)** changes in permeability, nutrient uptake and transporter activity in the hPB models that mimic data from in vivo studies. Barrier integrity studies confirmed the establishment of a robust barrier in our hPB utilizing both Tbs, especially in early-stage models, with values aligning with those reported in other studies on placenta in vitro models^11,55,56^, ex vivo placenta models^57^ and clinical investigations on pregnancy outcomes in women exposed to exogenous substances at different gestational periods^58^. This protective role of placenta also involves the prevention of hazardous agents from contacting the fetus. Our transcriptome analysis demonstrated an increase of genes encoding xenosensors in early-stage placenta models and those encoding drug-metabolizing enzymes in late-stages, with the latter also showing some significance at the protein level in the model obtained from the primary Tbs^59–62^. Our results from the drug efflux assays revealed significant variations in pump activity depending on the gestational stage: P-gp demonstrated substantially higher activity in early-stage models compared to late-stage models, consistent with its role as the predominant transporter in first-trimester placental tissue^63^; BCR-P expression and activity was variable, as has been reported^29,63^; and an increase in activity was seen from MRPs in late-stage models, as expected due to the elevated expression of most members of the MRP transporter in advanced stages^64–66^. This protective role also involves immune responses^67^. Our findings demonstrate substantial variations at transcript and protein levels of defense mechanism throughout different stages, influenced by the nature of the pathogenic insult, especially viral defense, which was markedly over-expressed in late stages across both cell lines. Furthermore, we noted an increase in cytokines throughout the later stages, corresponding with prior studies that reported cytokine activity increases with gestational age, supporting inflammatory state that initiates parturition^59,68^. Another key function of the human placental barrier is modulating the transfer of nutrients to the fetus, a multi-step process that involves both uptake into the trophoblast cells from the maternal side and subsequent transport across the barrier to the fetal circulation. Our study design allowed us to dissect these two critical steps. For that, we studied the transport of Glucose, free fatty acids (FAs) and amino acids, essential nutrients for adequate embryonic development. While our transcriptomic and proteomic analyses showed no significant differences in the expression levels of key glucose transporters between the early- and late-stage models, functional assays revealed distinct stage-dependent dynamics. Specifically, we observed a significant increase in both the uptake of glucose by the trophoblast layer and its subsequent directional, trans-placental transport to the fetal compartment in the late-stage models. We also observed that lipid transporters significantly increased in late-stage models, whereas protein levels showed little variation. Higher uptake of FA was observed in late-stage models, correlating with higher levels of expression and transport activity of maternal lipids into the placenta across the whole gestational process^69–72^. Our transcriptomics and metabolomics data also indicated a significant upregulation and activity of amino acid transporters in the late-stage model. Overall, all these results are indicative that our hPB models recapitulate the adaptative pattern of placenta to fetal nutritional requirements by showing a direct correlation between uptake-transfer of nutrients and gestational age.

While this work presents advanced placental models demonstrating key features of different gestational stages, it is crucial to acknowledge certain inherent limitations that frame the context of our findings and offer avenues for future investigation. A notable limitation of our ‘early-stage’ model is its reliance on trophoblast cells derived from term placentas. Although our biofabrication approach reproduces key morphological features and oxygen-dependent functional responses characteristic of the first trimester, term-derived cells are unlikely to fully mirror the developmental identity of early gestation trophoblasts, since they possess distinct epigenetic, transcriptional, and functional signatures that cannot be entirely recapitulated through environmental modulation alone. Therefore, integrating human induced pluripotent stem cell (hiPSC)-derived trophoblasts in future work would greatly enhance the model’s value for patient-specific studies and for investigating genetic placental disorders. Furthermore, sex as a biological variable is a critical determinant of placental physiology and pathology. Because the primary cytotrophoblasts, fibroblasts, and immortalized cell lines utilized in this study were derived exclusively from male donors, this single-sex sourcing may restrict the generalizability of our findings to female placentas. Given the well-documented sexual dimorphism in placental gene expression, metabolic regulation, and stress responses, future investigations utilizing the hPB platform must systematically incorporate female-derived cellular components to elucidate sex-specific mechanisms at the maternal-fetal interface. A degree of discordance was also noted between our multi-omics datasets and functional assays, a common challenge in systems biology that likely reflects the intricate layers of post-transcriptional regulation and the inherent biological variability between donors. This highlights the importance of a multimodal analytical framework and caution against overinterpretation of any single data type. Although our data demonstrate the intrinsic activation of defense pathways as a baseline functional hallmark of trophoblasts, the absence of maternal and immune compartments represents a notable limitation. In vivo, comprehensive placental defense mechanisms and disease pathogenesis rely heavily on complex immunological crosstalk. Currently, our hPB platform captures only the trophoblast- and stroma-mediated facets of these immune responses. To more comprehensively model the maternal-fetal crosstalk central to placental pathologies, subsequent studies will aim to integrate additional key cell types, including fetal macrophages (Hofbauer cells) and maternal immune and decidual cells. Finally, although the current investigation utilized a static culture system, the foundational methodology for directing the co-culture and differentiation of placental cells to emulate distinct trimesters possesses significant adaptability. This biofabrication strategy could be readily translated to dynamic microphysiological systems^73,74^, such as perfused organ-on-a-chip platforms, which would permit the introduction of hemodynamic shear stress and facilitate more sophisticated investigations into the mechanobiology of the maternal-fetal interface.

In conclusion, we have established an in vitro multi-layered, vascularized *3D* human placenta barrier model that mimics different stages of gestation and recapitulates the changes in functionality that have been reported in vivo. This hPB model includes relevant functional assays that are robust and reliable in a high troughput format. This is a hPB assay platform with broad applicability to investigate disorders of pregnancy, development of therapeutics, and drug safety assessment^2,75^, that is easily accessible to the broader biomedical community.

## Materials and methods

### Cell culture

Primary cytotrophoblast derived from healthy term placenta were obtained from The Amnion Foundation (Cat# 1230). We obtained CTBs for two donors: Donor 1 (male) and donor 2 (male). These cells were cultured on coated flasks with Matrigel 1% (Cat# 356234, Corning), and in CTBPRO2GRO™ culture media, containing 5% fetal bovine serum (FBS, Cat# 16140071, ThermoFisher), 0.1mM 2-Mercapto-ethanol, (Cat# 21985023, ThermoFisher) and 1% penicillin/streptomycin (Cat# 15140122, ThermoFisher). Immortalized cytotrophoblast cells derived from healthy term placenta were obtained from the laboratory of Dr. Ramkumar Menon at University of Texas Medical Branch^76^ from two donors, donor 1 (HM3, male) and donor 2 (HP1, male). Placental specimens used for this study were deidentified and considered as discarded human specimens that do not require institutional review board approval. Placental specimens were collected from John Sealy Hospital at the University of Texas Medical Branch at Galveston, Texas, USA, in accordance with the relevant guidelines and regulations of approved protocols for various studies (UTMB 11–251; University of Texas Medical Branch at Galveston).These cells were cultured on coated flasks (Matrigel 1%), in DMEM/F-12 medium supplemented with 0.1 mM 2-mercaptoethanol, 0.2% FBS, 1% penicillin/streptomycin, 0.3% BSA (Cat# A4737, Millipore Sigma), 1% ITS-X supplement (Cat# 51500056, ThermoFisher), 1.5 µg/mL l-ascorbic acid (Cat# A92902, Millipore Sigma), 50 ng/mL epidermal growth factor [EGF] (Cat# 236-EG, R&D Systems), 2 µM CHIR99021 (Cat# SML1046, Millipore Sigma), 0.5 µM A83-01 (Cat#SML0788, Millipore Sigma), 1 µM SB431542 (Cat# 616464, Millipore Sigma), 0.8 mM valproic acid (Cat# P6273, Millipore Sigma), and 5 µM Y27632 (Cat#S68-800, Millipore Sigma). GFP Expressing Human Umbilical Vein Endothelial Cells from term placenta (GFP-HUVECs, Cat# cAP-0001GFP) and Human Placental Microvascular Pericytes (HPlMVPCs, Cat# cAP-0029) from term placenta were obtained from Angioproteomie. GFP-HUVECs were cultured on coated flasks (fast coating solution, AngioProteomie), in VascuLife^®^ EnGS Endothelial Medium (Cat# LL-0002, LifeLine Cell Technology). HPlMVPCs were grown on coated flasks (fast coating solution, Cat#cAP-01, AngioProteomie) in Pericyte Growth Medium (Cat# cAP-09, Angioproteomie). Term placental Fibroblasts were obtained from The Amnion Foundation (Cat# 1255) and grown using FibroLife Medium (Cat# LL-0001, LifeLine Cell Technology). All cell lines were maintained in an incubator at 37 °C with 5% O2 and 5% CO2, and the media was changed every other day. The cells were dissociated at 70% confluency and sub-cultured using TripleExpress (Cat# 12604013, ThermoFisher).

### Biofabrication of placenta tissue models at different gestational stages of pregnancy

The biofabrication of the vascularized hPB involves two steps: the creation of the trophoblast layer and the bioprinting of placental stroma layer. To develop the placenta stroma, we initially printed an O-rings (2.65 mm radius; 0.7 mm height) utilizing GelMA (Cat# 900628, Millipore Sigma) hydrogel, which was loaded in a 10 ml syringe fitted with a 21G needle (inner diameter of 0.53 mm) at 16 °C in a RegenHU bioprinter (RegenHU Ltd., Switzerland; R-GEN 200) on the rim of the basal side of a 96-well transwell insert (Cat# 3392, Corning), the well surfaces of which were pre-treated with O_2_-plasma. The O-rings were subsequently crosslinked utilizing UV light (Phoseon’s KeyPro™ KP100 microplate system) and allowed to dry overnight. GFP-HUVECs, placenta fibroblasts, and HPlMVPCs were combined in a 7:1:0.7 (million cells/ml) ratio within a fibrin-based hydrogel [3 mg/ml Fibrinogen (Cat# F3879, Millipore Sigma) and 6% w/v Porcine Gelatin (Cat# G1890, Millipore Sigma) in PBS] and bioprinted in a specific ring-shaped pattern using a 5 ml syringe and a 21G needle on the opposing side of pre-printed 96-well transwell inserts (1.35 mm radius; 0.7 mm height), whose surface was previously treated with O2-plasma. This spatial configuration was specifically designed to allow for the simultaneous measurement of both vasculogenesis and angiogenesis. Subsequently, 40 µl of acellular gel, consisting of 5 mg/ml Fibrinogen and 1 U/ml Thrombin (Cat# T6884-1KU, Millipore Sigma), was applied to the basal side of the 96-well transwell insert surface to cover the unprinted region. The plate was incubated at room temperature for 15 min to harden the gel. After the acellular gel was properly crosslinked, 160 µl of vascular medium with 1 U/ml thrombin was added to each well of the receiver transwell plate, and the plate was incubated at room temperature for 1 h to allow fibrin gel formation. The vascular media was formulated by combining VascuLife culture media (containing 5% CDI iCell supplement) and RPE media [modified Eagle’s alpha-modified medium (Cat# 32561102, ThermoFisher) supplemented with 1% v/v N-2 (Cat# 17502048, ThermoFisher), glutamine (Cat# 25030081, ThermoFisher), 1% penicillin and streptomycin, 1x non-essential amino acids (Cat# 11140050, ThermoFisher), 125 mg taurine (Cat# T8691, Millipore Sigma), 10 µg hydrocortisone (Cat# H0888, Millipore Sigma), and 0.0065 µg triiodothyronine (Cat# T6397, Millipore Sigma) cocktail dissolved in PBS] in a 1:1 ratio, along with the addition of VEGF (21.25 ng/ml; Cat#: 293-VE-500, R&D Systems), Recombinant Human Angiopoietin-1 Protein (ANG1, 100 ng/ml, Cat#: 923-AN-025, R&D Systems), and aprotinin (0.075 U/ml, Cat#: J11388.MC, ThermoFisher). Bioprinted transwell inserts were incubated overnight at 37 °C with 5% O2 and 5% CO2. Media change was performed on Day 1 to eliminate the thrombin and was renewed every other day.

The trophoblast layer was created by seeding a bilayer of cytotrophoblasts (CTB) on the apical side of the transwell membrane. For that, we seeded a first layer of 100 ml of CTB suspension (5 × 10^5^ cells/ml in growth media, 270 K cells/cm^2^ (first layer) and a second layer of CTB at the same density, after 24 h. The day after the second layer was seeded, we exposed the CTB bilayer to differentiation basal media [DMEM/F12 (Cat#: 11320033, ThermoFisher) supplemented with 0.1 mM 2-mercaptoethanol, 0.5% penicillin/streptomycin, 0.3% BSA, 1% ITS-X supplement, 2.5 µM Y27632, 4% knockout serum replacement (Cat#: 10828028, ThermoFisher)] supplemented with 25µM Forskolin^77^ (Cat# F6886, Millipore Sigma) and hypoxic conditions (5% O_2_) for 4 days. To recreate the early-stage model, the pre-differentiated hPB tissues were exposed to hypoxic conditions for 7 more days. Conversely, to recreate the late-stage model, the pre-differentiated hPB tissues were exposed to normal oxygen conditions (21% O_2_) for 7 more days, under differentiation basal media. During the maturation culture time (7 days at specific conditions of oxygen), the apical compartment (trophoblasts bilayer) of the hPB tissues models were maintained in complete differentiation media and the basal compartment (placental stroma layer) in vascular media. The step-by-step protocol of hPB tissue models’ formation is illustrated in Fig. 1B.

### Immunofluorescence

Placenta tissue models were fixed with 4% paraformaldehyde (PFA, Cat# 15714-S, Electron Microscopy Sciences) for 20 min at RT. For fluorescent observation, fixed hPB tissues were treated with 5% goat serum (Cat# ab7481, Abcam), and 0.25% Triton-X (PI85111, Thermo Fisher) for 1 h. The tissues were incubated overnight with the primary antibodies: Cytokeratin 7 (CK7) antibody (1:100, Cat# AB68459, Abcam); Chorionic gonadotrophin (hCG) antibody (1:100, Cat# AB187285, Abcam); Zonula Occludens-1 (ZO-1) antibody (1:50, Cat# 33–9100, Thermo Fisher). After washing steps, the secondary antibodies (1:500) and/or Alexa 488 Conjugated Phalloidin (1:600, Cat# A12379, Thermo Fisher) were added to the tissues and incubated for 45 min at room temperature. Nuclei were labeled using Hoescht 33,342 (Cat# 62249, Thermo Fisher) after the secondary antibody incubation. Control staining was performed with secondary antibodies only to exclude unspecific antibody reaction. Following staining, the tissues were carefully removed from the transwell platform and mounted onto a coverslip. For histological observation, fixed tissues were processed into 10 μm thick Optimal Cutting Temperature (OCT)-embedded cryo-sections. The immunostaining for CK7 and hCG were performed following the protocol described above. Cryosectioned samples and whole tissue samples were mounted using Fluoromount G (Cat# 0100-01, Southern Biotech,). Images were acquired using a confocal microscope (Nikon Elipse Ti2). Image processing and three-dimensional rendering were carried out using Nikon software.

### Vasculogenesis and angiogenesis analysis of placenta stroma

Fluorescence cell imaging was performed using the Opera Phenix High-Content Screening System (Harmony v5.2, Revvity), with images acquired using a 5× objective in confocal mode (z = 470 μm, 50 μm per stack). Maximum intensity projections of entire wells and plates were reconstructed and analyzed in SImA (Signal Image Artist v1.4.1, Revvity). Vascular areas and angiogenic tube-like structures were segmented from GFP fluorescence of HUVECs (Fig. 2N). Skeleton regions, representing the central paths of the segmented vasculature, were extracted using SImA’s built-in skeletonization tools for quantitative tube morphology analysis. Customized Angiogenesis Assay–Specific Building Blocks (Revvity) were applied to identify vessel-like structures. Morphological features—including vascular area, tube length, width, and the number of nodes and tubes—were then quantified (Fig. 2P and Supplementary Fig. 2 A, B).

### Transcriptomic analysis

RNA of hPB tissue models (3 replicates for each hPB tissue models) were extracted using Trizol (Zymo, R2050-1) and isolated using Direct-zol-96 RNA Direct-zol-96 RNA, according to the manufacturer’s instructions. RNA was quantified by measuring absorbance at 260 nm, and RNA purity was determined by the ratios OD260 nm/280 nm and OD230 nm/280 nm using the NanoDrop instrument (DeNovix DS-11). Library preparation and sequencing were performed at the NHLBI DNA Sequencing and Genomics Core. Sequencing was conducted on the NovaSeqX platform using 2× 100 bp paired-end reads, generating 40–50 million reads per sample. Raw RNA-seq reads were aligned to the human reference genome (hgCh38) using the STAR aligner. Gene-level read counts were obtained using HTSeq. Differential expression analysis was performed in R using the DESeq2 package^78^, with raw count data as input. Genes with fewer than 10 total counts across all samples were excluded. Normalization was conducted using internal median-of-ratios approach from DESeq2 to adjust for variability in library size across samples. A negative binomial generalized linear model was applied to estimate dispersion and test for differential expression between two developmental stages for each cell line. Principal component analysis (PCA) was performed to evaluate the separation of samples across different stages, and volcano plots were generated to visualize differentially expressed genes. Genes with an adjusted p-value (Benjamini–Hochberg FDR) < 0.05 and an absolute log2 fold change > 1 were considered significantly differentially expressed compared between different stages of hPB tissue models.

### Proteomic analysis

The hPB tissue models were suspended in NP40 buffer containing complete protease inhibitor at 4 °C overnight, followed by ultrasonication for 2 min on ice. The lysates were then centrifuged for 20 min at 15,000 g, and supernatant was collected in a fresh tube for further protein concentration measurement using the bicinchoninic acid (BCA) method. Proteins from different tissue models were denatured and reduced with the addition of 0.1% RapidGest (Waters Corporation) and 15 mM Tris(2-carboxyethyl)phosphine (TCEP, Millipore Sigma) at 55 °C for 45 min, and alkylated with 15 mM iodoacetamide (IAA, Millipore Sigma) in the dark for 30 min. The alkylated proteins were then precipitated by adding 9:1 (v/v, acetonitrile/protein sample) of cold acetonitrile (ACN, Thermo Scientific, −20 °C) and incubating for 15 min on ice. After aspirating the supernatant, the proteins were redissolved in trypsin/Lys-C (Promega) buffer in 25 mM NH4HCO3 (Millipore Sigma, pH 8.0) at an enzyme/substrate ratio (m/m) of 1:50 and incubated at 37 °C overnight. The digestion was quenched by adding 1% of formic acid (FA, Fisher Chemical), and the digests were collected by centrifugation for further LC- MS/MS analysis. LC-MS/MS analysis of the protein digest was performed using an UltiMate 3000-nano LC system coupled to the Orbitrap Fusion Lumos Tribrid mass spectrometer equipped with the Nanospray Flex ion source. Peptides were loaded onto the trap column (AcclaimPepMap 100 C18, 75 μm × 2 cm, particle size: 3 μm, 100 Å) by autosampler using loading solvent (2% ACN in 98% MS-grade water, 0.1%FA) at a flow rate of 4 µL/min. Elution of peptides from the analytical column (Aurora Ultimate XT 25 cm × 75 μm, 1.7 μm, C18; IonOpticks, Australia) was performed using a 120 min method (99 min gradient) starting at 98% buffer A (0.1% FA in MS-grade water) at a flow rate of 400 nL/min. The mobile phase was maintained at 2% buffer B (80% ACN, 19.9% water, 0.1% FA) for 5 min, 2–6% B for 1 min, 6–31% B for 76 min, 31–43% B for 13 min, 43–95% B for 1 min, and maintained at 95% B for 8 min, followed by re-equilibration of the column with 2% B for 15 min. Column oven temperature was set as 50 °C. The mass spectrometer was operated in positive-ionization mode with the Nanospray Flex ion source with spray voltage set at 1800 V, and ion transfer tube temperature set at 300 °C. The MS scan was operated at data-independent acquisition mode, with a precursor range of 390–1010 m/z and an isolation window of 8 m/z with 1 m/z overlap, resulting in 75 windows for each scan cycle. The resolution for MS1 scan was set to 120 K. Both standard AGC target and auto maximum injection time were selected. The MS2 fragmentation was performed in Orbitrap with 30 K resolution and a normalized collision energy of 30% at HCD activation mode. The AGC target was set to 800%. The MS2 scan range was defined as 145–1450 m/z, and the loop control was set to 3 s. Proteome Discoverer software (version 3.1.1.93, Thermo Fisher) was used for peptide identification and quantitation. Searches were performed using CHIMERYS v2.7.17 with inferys 3.0.0 fragmentation prediction model. The MS raw data were searched against a Swiss-Prot human database (version Nov 2022, reviewed database) consisting of 20,328 entries using the following parameters: Max. fragment mass tolerance was set as 20 ppm; up to two miss cleavages was set for trypsin digestion; fixed modification considered was carbamidomethylation of cysteine; variable modification considered was oxidation of methionine residues. Peptide identification false discovery rates (FDR) were limited to a maximum of 0.01. Label-free quantification analysis used the “Fragment Ions Quantifier” node from Proteome Discoverer.

The same significance thresholds used for the RNA-seq data were applied to the proteomics dataset. Proteins with an adjusted p-value (Benjamini–Hochberg false discovery rate) < 0.05 and an absolute log₂ fold change (the ratio of protein abundance) > 1 were considered significantly differentially expressed between the different stages of the hPB tissue models. Principal component analysis (PCA) and volcano plots were also generated using the same methods applied to the RNAseq dataset, to assess stage-specific sample separation and identify differentially expressed proteins.

### Transepithelial Electrical Resistance (TEER) measurements

Placental barrier integrity was measured with an automated TEER measurement system (World Precision Instruments, Sarasota, FL, United States) containing 300 µL of medium on the basolateral side and 100 µL of medium on the apical side of transwell. Transendothelial electrical resistance (TEER) values were corrected for the surface area (Ω・cm2).

### Permeability assays

Barrier function of the hPB tissues models was assessed by measuring the transport of fluorescent molecules of different weight, 457 Da Lucifer Yellow (LY, Cat# L0144, Millipore Sigma) and 3 kDa Dextran (DT) Tetramethylrhodamine (TAMRA) (Cat# D3307, Millipore Sigma), between the apical (maternal side) and basal compartments (fetal side). LY and TAMRA-DX (0.1 mg mL − 1 in DMEM/F-12 media) was introduced to the apical compartment and incubated for 1, 3, 6 and 24 h. Volumes of 20 µL were sampled from the media at the basal compartment at every time point and the fluorescence intensity of the collected samples was measured together with a standard dilution series of the respective molecules using a microplate reader (Tecan) at emission/excitation of 580/620 for DT and 485/520 nm for LY. The permeated amount of fluorescent molecules at a time point was normalized to the membrane without tissue. The percentage rate of permeability was calculated using the following equation:

$$\:\%\:Permeability=\left(\frac{{C}_{t}}{{C}_{m}}\right)\times\:100$$

where C*t* and C*m* denote for concentration (µg/ml) of fluorescent molecules in the basal compartment of the tissue models and bare membrane at specific time point, respectively. At least three biologically independent replicates for each condition and time point were tested.

### Assessment of nutrient uptake by trophoblast layer of hPB models

The absorption of nutrients, specifically glucose and fatty acids, by the trophoblast layer of the hPB tissue models was assessed using Glucose-Cy5 and C12-BODIPY, respectively. Those nutrients labeled with distinct fluorophores were added to the apical compartment in the basal differentiation medium at a concentration of 50nM (Glucose-Cy5, Cat#SML3233, Millipore Sigma) and 10nM (C12-BODIPY, Cat#SML4395, Millipore Sigma). The medium in the basal compartment was replaced with low glucose DMEM (Cat#11966025, ThermoFisher). The hPB tissue models were thereafter incubated for one hour at 37 °C under specific oxygen conditions. Fluorescence imaging was conducted using an Opera Phenix High-Content Screening System. Images were captured using a 5× objective in confocal acquisition mode, and maximum intensity projections were analyzed with Harmony software (PerkinElmer).

The activity of nutrients transporters for glucose and fatty acids was analyzed by using inhibitors of specific intake pathways. For glucose, selective inhibitors of the main glucose transporter in placenta, glucose transporter (GLUT) 1, including GLUT1 inhibitor 1 (5, 10, 25µM; Cat# HY-139605, MedChemExpress), STF-31 (2,5, 10µM; Cat# HY-18728, MedChemExpress), and WZB117 (5, 10, 25µM; Cat# HY-19331, MedChemExpress), as well as competitive inhibitors of GLUT-1, 2, 3 and 4, such as KL-11,743 (0.5, 1, 2µM; Cat# HY-145597, MedChemExpress), and BAY-876 (2, 5, 10µM; Cat# HY-100017, MedChemExpress). For fatty acids, candidate inhibitors included Nortriptyline (5, 20, 40µM; Cat# HY-118620, MedChemExpress), EIPA (10, 20, 40µM; Cat# HY-101840, MedChemExpress), and lipofermata (5, 20, 40µM; Cat# HY-116788, MedChemExpress). Nortriptyline and EIPA inhibits fatty acid uptake by suppressing macropinocytosis, a main route for fatty acid entry; lipofermata inhibits fatty acid uptake by specifically attenuating FATP2-mediated fatty acid transport without impacting other cellular functions. The concentrations tested were selected based on IC50 or on in dose-window previously tested in in vitro studies^81–85^.

To compare the hPB tissue models, we measured the % activity using the following equation:

$$\:\%\:Activity=\left(\frac{{{RFU}_{-inh}-RFU}_{+inh}}{{RFU}_{-inh}}\right)\times\:100$$

where RFU*-inh* and RFU *+ inh* denote for Relative Fluorescence Unit mean values of tissue samples without and with incubation with nutrient inhibitor, respectively. At least three biologically independent replicates for each condition and time point were tested.

### Evaluation of nutrients transport of hPB models by HPLC-QQQ multiple reaction monitoring (MRM)

To analyze glucose transport across the hPB to the fetal side, the apical compartment was filled with differentiation basal medium or differentiation medium containing 10 mM D-glucose (Cat#G8270, Millipore Sigma), 200 µM L-leucine (Cat#L8912, Millipore Sigma) and 5.3 mg/L linoleic acid (Cat#L5900, Millipore Sigma). The basal chamber (fetal side) was filled with differentiation basal medium. Media samples from the basal chamber were collected over a period of 24 h. Three biologically independent replicates for each condition were tested. Liquid chromatography was performed on an Agilent 1290 Infinity II LC system (Agilent Technologies, Wilmington, DE, U.S.A.) equipped with a binary pump, multicolumn thermostat, and autosampler. The mobile phases used for the separation were MS-grade water with 0.1% formic acid (solvent A) and MS-grade acetonitrile with 0.1% formic acid (solvent B). For reversed phase (column: Waters BEH, C18, 1.7 µM, 2.1*50 mm), gradient elution of linoleic acid from the analytical column was performed using a gradient starting at 2% B at a flow rate of 0.4 mL/min. The mobile phase was held at 2% B for 0.2 min, then going to 95% B for 2.8 min, and maintained at 95% B for 0.8 min, followed by 2% B for 0.2 min and re-equilibration of column with 15% B for 0.5 min. Separations were performed at a column temperature of 35 °C with a total run time of 4.5 min. For hydrophilic interaction liquid chromatography (HILIC column: HALO, HILIC, 2.7 µM, 2.1*50 mm), gradient elution of glucose and leucine from the analytical column was performed using a gradient starting at 95% B at a flow rate of 0.4 mL/min. The mobile phase was held at 95% B for 0.2 min, then going to 50% B for 2.8 min, 5% B for 0.8 min, followed by 95% B for 0.2 min and re-equilibration of column with 15% B for 0.5 min. Separations were performed at a column temperature of 35 °C with a total run time of 4.5 min. A varying volume of sample was injected onto the column for each experimental run. Agilent 6470 triple quadrupole mass spectrometer (Agilent, Santa Clara, CA, U.S.A.) equipped with a Jet Stream electrospray ionization (AJS ESI) interface source was used for the multiple reaction monitoring (MRM) method development. Before use, the instrument was tuned and calibrated in positive mode. The gas temperature, gas flow, sheath gas temperature, and sheath gas flow were set to 325 °C, 10 L/min, 375 °C, and 11 L/min, respectively. Electrical voltages were optimized for the capillary voltage at + 3500 V, nebulizer voltage at + 1000 V. To achieve the best selectivity and sensitivity, the mass spectrometer was operated in MRM mode for the quantification analysis with details shown in Table 1.

**Table 1:** Parental ion and product ion for quantification analysis of linoleic acid, glucose and leucine.

Compound Name	Prec Ion	MS1 Res	Prod Ion	MS2 Res	Dwell (ms)	Frag (V)	CE (V)	Cell Acc (V)	Polarity
Linoleic acid	263.1	Wide	245.2	Wide	10	135	9	5	Positive
Glucose	203.1	Unit	203	Unit	20	135	1	4	Positive
Leucine	132.1	Unit	86.1	Unit	20	80	8	4	Positive

Note: Prec: precursor; Res: Resolution; Prod: product; Frag: fragment; CE: collision energy; Acc: accelerator.

### Drug efflux activity assay

The activity of major drug efflux transporters, including P-glycoprotein (P-gp), the multi-drug resistance protein (MRP) family, and Breast Cancer Resistance Protein (BCR-P), in the hPB tissue models were evaluated by measuring the efflux of fluorescent substrates specific to each transporter, after exposure to differentiation basal media with or without drug transporter inhibitor. Rhodamine 123 (Rho123; 5.25 µM; Cat# R302, Millipore Sigma) was used as the fluorescent dye to be the substrate of P-gp and Cyclosporine A (CSA, Cat#30024, Millipore Sigma), as the inhibitor^79^. 5-chloromethylfluorescein diacetate (CMFDA; 5 µM, Cat# C2925, ThermoFisher) as the specific substrate for MRPs and MK571 (Cat# M7571, Millipore Sigma) as an inhibitor^80–82^. BODIPY-Prazosin (250 nM, Cat# B7433, ThermoFisher) was used as the substrate of BCR-P and ko-143^89^ as the inhibitor (Cat# HY-10010, MedChemExpress). The concentrations of inhibitors tested were 5, 10 and 50µM in all the cases. The candidate inhibitors and concentrations were selected based on previously tested in in vitro studies^82–85^. Fluorescent substrates mixed with or without inhibitor at specific concentrations in differentiation basal media and added to the apical side of the hPB models, and incubated for 1 h at 37 and subsequently imaged using an Opera Phenix High-Content Screening System. Images were captured using a 5× objective in confocal acquisition mode, and maximum intensity projections were analyzed with Harmony software (PerkinElmer).

To compare the hPB tissue models, we measured the % efflux transporter activity using the following equation:

$$\:\%\:Activity=\left(\frac{{{RFU}_{+inh}-RFU}_{-inh}}{{RFU}_{+inh}}\right)\times\:100$$

where RFU*-inh* and RFU *+ inh* denote for Relative Fluorescence Unit mean values of tissue samples without and with incubation with transporter inhibitor, respectively. At least three biologically independent replicates for each condition and time point were tested.

### Enzyme-Linked Immunosorbent Assays (ELISA)

Supernatants from hPB models at day 7 after differentiation under specific oxygen conditions were collected to determine the secretion levels of placenta-relevant hormones by enzyme-linked immunosorbent assay (ELISA). Human chorionic gonadotropin (hCG) was detected using Human hCG ELISA Kit (Abcam, ab100533). Progesterone was detected using Progesterone Competitive ELISA Kit (ThermoFisher scientific, EIAP4C21). Human Estradiol was detected using Human Estradiol E2 ELISA Kit (ThermoFisher scientific, KAQ0622). Placental Lactogen (PL) was detected using Human PL ELISA Kit (Biomatrik, EKE62865-96T). All kits were used according to the manufacturer’s protocol. Absorbance was measured via microplate reader.

### Statistical analysis

Statistical analysis and graphical representation were performed in GraphPad Prism 8.3.0 (Graphpad, San Diego, CA, USA). Data are presented as means ± standard errors of the means (SEMs). Comparisons were made among groups by using Student’s t-tests or a 2-way ANOVA followed by Sidak’s post hoc analysis. *P* < 0.05 was considered as statistically significant. For all statistical tests, ns indicates *p* > 0.05, *indicates *p* < 0.05, ** indicates *p* < 0.01, *** indicates *p* < 0.001, *** indicates *p* < 0.0001. Each experiment was repeated at least three times.

## Supplementary Information

Below is the link to the electronic supplementary material.

Supplementary Material 1

Supplementary Material 2

Supplementary Material 3

Supplementary Material 4

Supplementary Material 5

Supplementary Material 6

Supplementary Material 7

Supplementary Material 8

Supplementary Material 9

Supplementary Material 10

Supplementary Material 11

Supplementary Material 12

Supplementary Material 13

Supplementary Material 14

Supplementary Material 15

Supplementary Material 16

## Data Availability

The transcriptomics datasets generated and analyzed during the current study is available in the GEO repository [GSE318016]. The mass spectrometry proteomics data have been deposited to the ProteomeXchange Consortium via the PRIDE 86 partner repository with the dataset identifier PXD072837.
