# Supplementary material for: Recapitulating gestational progression: a high-throughput 3D bioprinted vascularized human placenta demonstrating stage-specific molecular and functional signatures of human pregnancy
Source: Sci Rep. 2026 Jul 28;16:24140. doi: 10.1038/s41598-026-61708-y (PMC13443107; doi:10.1038/s41598-026-61708-y)
Supplement: Supplementary file 1 [file 41598_2026_61708_MOESM16_ESM.docx]

**Supplementary Fig. 1:** Structural analysis of human placenta 3D models at different stages of gestation (Donor 2). A, D, G, H) Immunofluorescence images obtained from confocal microscopy of the trophoblast bilayer in human placenta models, utilizing trophoblast marker (CK7), syncytiotrophoblast marker (CGH), and nuclear marker (DAPI). B, E, I, K) Histological slices of human placenta models stained with markers for the trophoblast layer (CK7, CGH) and the stroma layer (CD31). 3D representations from Z-stack projection of human placenta models by confocal fluorescence imaging (C, F, J, L). Scale bar: 100µm.

## **Supplementary Fig. 2:** Quantitative tube morphology analysis of the placental stroma from human placenta 3D models at different stages of gestation. A) Vascular areas and angiogenic tube-like structures detection and segmentation process from GFP fluorescence of HUVECs by SImA’s built-in skeletonization tool. B) Morphological features including vascular area, vessel tube and nodes identified by Customized Angiogenesis Assay–Specific Building Blocks. Scale bar: 200µm.

**Supplementary Fig. 3:** The enrichment score (ES) plots for all differentially expressed genes related to selected biological processes between human placenta models using primary (A) and immortalized (B) cell sources.

## **Supplementary Fig. 4:** Comparison of transcriptomic and proteomic profiles of human placenta models at different stages of gestation, using primary cell sources. (A) Venn diagram showing the overlapping of differentially expressed genes and their corresponding proteins between human placenta models. (B-H) Box plots representing the differentially expressed genes and their corresponding proteins involved in selected biological processes, between gestational stages of placenta model.

## **Supplementary Fig. 5:** Comparison of transcriptomic and proteomic profiles of human placenta models at different stages of gestation, utilizing immortalized cell sources. (A) Venn diagram illustrating the overlapping of differentially expressed genes and their corresponding proteins between human placenta models. (B-H) Box plots showing the differentially expressed genes and their corresponding proteins involved in selected biological processes, across gestational stages of the placenta model.

**Supplementary Fig. 6:** Tight junction protein ZO-1 and F-actin immunofluorescent staining images of trophoblast layer in placenta models (donor 2) after maturation culture time. Scale bar: 100µm.

**Supplementary Fig. 7:** Nutrients transport and drug efflux activity of human placenta 3D models changed at different stages of gestation (Donor 2). (A and E) Confocal fluorescence imaging of Glucose-Cy5 uptake by the trophoblasts layer of the human placenta models at different gestational stages. (C and G) Nutrient uptake activity by main transporter of glucose (GLUT1). (B and F) Confocal fluorescence imaging of C12-BODIPY uptake by the trophoblasts layer of the human placenta models at different gestational stages. (D and H) Nutrient uptake activity by main fatty acid transporter (FATP2). (I and J) Confocal images of trophoblasts from human placenta 3D models at different gestational stages containing fluorescent substrate compounds of P-gp, MRPs and BCR-P pumps (green), after incubation with and without specific inhibitors for P-gp, MRPs and BCR-P pumps. (K and L) Transporter activity of P-gp, MRPs and BCR-P pumps in trophoblasts from human placenta 3D models at different gestational stages. Scale bar:200μm. Each experiment was done in biological replicate (N=3), error bars represent standard deviation. Statistical significance was determined by two-way ANOVA for nutrient uptake and transfer values, and efflux activity differences between placental stage models and Multiple unpaired t-test for nutrient uptake activity differences between placental stage models. (**** P < 0.0001,*** P <0.001, ** P < 0.01, * P < 0.05, ns P >0.05).

**Supplementary Fig. 8:** Intensity measurements of fluorescent substances in the trophoblast layer of the human placenta models at different gestational stages, both with and without inhibitor treatment for nutrients uptake and drug efflux assays. (A-D) Fluorescence intensity values of Glucose-Cy5 in the trophoblasts layer of the human placenta models at different gestational stages, with and without GLUT inhibitor treatment (GLUT1-IN-1). (E-H) Fluorescence intensity values of C12-BODIPY in the trophoblasts layer of the human placenta models at different gestational stages, with and without FA transport inhibitor treatment (Lipofermata). Fluorescence intensity values of fluorescent substrate compounds of P-gp (I-L), MRPs (M-P) and BCR-P (Q-T) pumps in the trophoblasts layer of the placenta models after incubation with and without specific inhibitors for P-gp (Cyclosporine A), MRPs (MK571) and BCR-P (ko-143) pumps . Each experiment was done in biological replicate (N=3), error bars represent standard deviation. Statistical significance was determined by two-way ANOVA for fluorescence value and efflux activity differences between placental stage models (**** P < 0.0001,*** P < 0.001, ** P < 0.01, * P < 0.05, ns P >0.05).

**Supplementary Fig. 9:** Evaluation of candidates for glucose transport inhibition in human placenta 3D models at different stages of gestation. (A and D) Confocal fluorescence imaging of Glucose-cy5 uptake by the trophoblasts layer of the human placenta models at different gestational stages. (B, C, E, F) Fluorescence intensity values of glucose-cy5 in the trophoblasts layer of the placenta models exposed to selective inhibitors of main glucose transporters in placenta at specific dose concentrations. Each experiment was done in biological replicate (N=3), error bars represent standard deviation. Scale bar:200μm. C1: Concentration 1; C2: Concentration 2; C3: Concentration 3; C4: Concentration 4 . Statistical significance was determined by two-way ANOVA for fluorescence intensity value differences between placenta models exposed to selective inhibitors compared to non-exposed placenta models (**** P < 0.0001,*** P < 0.001, ** P < 0.01, * P < 0.05).

## **Supplementary Fig. 10:** Evaluation of candidates for fatty acids transport inhibition in human placenta 3D models at different stages of gestation. (A and D) Confocal fluorescence imaging of C12-BODIPY uptake by the trophoblasts layer of the human placenta models at different gestational stages. (B, C, E, F) Fluorescence intensity values of C12-BODIPY in the trophoblasts layer of the placenta models exposed to selective inhibitors of fatty acids transport at specific dose concentrations. Each experiment was done in biological replicate (N=3), error bars represent standard deviation. Scale bar:200μm. C1: Concentration 1; C2: Concentration 2; C3: Concentration 3; C4: Concentration 4. Statistical significance was determined by two-way ANOVA for fluorescence intensity value differences between placenta models exposed to selective inhibitors compared to non-exposed placenta models (**** P < 0.0001,*** P < 0.001, ** P < 0.01, * P < 0.05).

## **Supplementary Fig. 11:** Evaluation of candidates for drug efflux transport inhibition in human placenta 3D models at different stages of gestation. Fluorescence intensity values of fluorescent substrate compounds of P-gp (A, E), MRPs (B, F) and BCR-P (C, G) pumps in the trophoblasts layer of the placenta models after incubation with and without specific inhibitors for P-gp, MRPs and BCR-P pumps, at specific dose concentrations. (D and H) Confocal fluorescence imaging of trophoblasts from human placenta 3D models at different gestational stages containing fluorescent substrate compounds of P-gp, MRPs and BCR-P pumps (green), after incubation with and without specific inhibitors for P-gp, MRPs and BCR-P pumps, at specific dose concentrations. Each experiment was done in biological replicate (N=3), error bars represent standard deviation. Scale bar:200μm. Statistical significance was determined by two-way ANOVA for fluorescence intensity value differences between placenta models exposed to selective inhibitors compared to non-exposed placenta models (**** P < 0.0001,*** P < 0.001, ** P < 0.01, * P < 0.05).

Supplementary Figure 1

Supplementary Figure 2

**Filter process**

**Analysis**

**Raw data**

**Early**

**Late**

**A**

**B**

**Vasculature tube**

**Skeleton**

**Tubes**

**Nodes**

**Early**

**Late**

Supplementary Figure 3

**A**

**B**

Supplementary Figure 4

Supplementary Figure 5

Supplementary Figure 6

Supplementary Figure 7

Supplementary Figure 8

Supplementary Figure 9

Supplementary Figure 10

Supplementary Figure 11
