# Supplementary material for: Recapitulating gestational progression: a high-throughput 3D bioprinted vascularized human placenta demonstrating stage-specific molecular and functional signatures of human pregnancy
Source: Sci Rep. 2026 Jul 28;16:24140. doi: 10.1038/s41598-026-61708-y (PMC13443107; doi:10.1038/s41598-026-61708-y)
Supplement: Supplementary file 2 [file 41598_2026_61708_MOESM1_ESM.docx]

**Supplementary Note 1**

The cluster of genes linked to the GO keywords “glucose metabolism”, “amino acid metabolism”, and “DNA biosynthetic process” show variations in metabolic activity across stages of hPB models. Genes associated with glycolysis were especially enriched in the early-stage models generated from both cell lines. Various key regulatory enzymes (i.e. GPI, PKM, PGKP, PFKP) and members of the enolase and aldolase families are among the most significantly over-expressed genes. Interestingly, a notable increase in the expression of genes associated with amino acid metabolism was mostly detected in the late-stage model derived from primary cells.

The DEG analysis also identified clusters of genes related to organism defense against xenobiotics, a critical function of the placenta barrier. Within the genes associated with responses to abiotic stimuli ^86^, an increase in the expression of xenosensors (AHR and SENSN2) was noted in the early-stage models. Genes related to the metabolism of pharmaceuticals and toxins (CYPs, GTSs, and SULTs) were primarily upregulated in the later-stage models. The expression of several drug efflux transporters varied considerably across stages. Other genes regulating antioxidant activity, were also among the most significantly elevated in late-stage placental models. The defense response to biotic stimuli was mostly associated with genes related to immune response ^87-89^. In the early-stage models, genes associated with antibacterial defense were notably overexpressed, and genes related to viral defense were significantly elevated in the late-stage models. Among the most upregulated genes encoding proteins with antibacterial activity (e.a.TLR4, H2Bs, SLPI and Elafin (PI3)). Genes associated with interferon production and signaling, including TLR3, DDX60 and members of the IFIT family, or GTPases (MX1 and MX2). Furthermore, genes encoding inflammatory cytokines essential for the recruitment of immune cells, including CXCLs, and CCL20, were substantially overexpressed in late-stage models, as well.

As a barrier between mother and fetus, the placenta plays an important role in the transfer of nutrients. In the cluster of differentially expressed genes associated with nutrient transport, major changes were observed in the genes related to lipid transport ^28,90^. Among them, those coding for lipids transporters such as ATP-binding cassette (ABC) transporters, fatty acid transport proteins (FATPs), fatty acid binding proteins (FABPs), apoliproteins and phospholipid transporters (PCTP, MFSD2), were mostly upregulated in the late-stage models. Transcripts associated with glucose transport exhibited small differences across stages, including the main glucose transporter in placenta, GLUT-1 ^28,90^. Transcripts with higher expression in the early-stage placenta model included those encoding proteins involved in iron trafficking and storage (i.e., TFR2, FTH1). The sub-cluster of transcripts associated with amino acid transport ^69,90-92^ including solute carrier (SLC) transporters were overexpressed in the late-stage placenta model assembled with primary Tbs. Other differentially expressed transporters included LAT-1 (SLC7A5) in the early-stage and SNAT-1 (SLC38A1) in the later stage.

In the cluster of deferentially expressed genes associated with placental hormone activity ^93^, the largest changes between placenta models at the two gestation stages were in the genes encoding for chorionic gonadotropin (CGBs) and placental lactogen (CSHs), which were notably upregulated in the late-stage models, for both primary and immortalized placenta models. Also elevated in late-stage models were transcripts encoding enzymes involved in the biosynthesis of estrogens.  In addition to the primary hormones, other genes associated with female pregnancy including pregnancy-specific glycoproteins (PSGs), which were significantly elevated in late-stage models.

Differences were also identified in transcripts regulating extracellular matrix (ECM) remodeling. In particular, the early-stage models exhibited high expression of structural components necessary for placentation, including collagens and laminins. Conversely, the late-stage models exhibited an upregulation of transcripts encoding proteases that are essential for the growth of the placenta in the maternal tissue, including Matrix Metalloproteinases (MMPs) and A disintegrin and metalloproteinase with thrombospondin motifs (ADAMTS). These data support the preparation of the placenta for parturition where the MMP activities are reported to higher promoting collagenolytic process. Vasculogenesis and angiogenesis are critical for successful placental maintenance and function. Differential expression of angiogenesis-related genes ^94,95^ was observed between the two developmental stages. ANGPTL4 was among the most up-regulated genes in early-stage placenta model. Genes involved in signaling pathways of vascularization ^95,96^ (e.g. PIK3Cs), vasculature maturation (e.g. ITGAV, NRCAM), and vascular tone (e.g. CALCRL, S1PR1, ADM) were found to be highly upregulated in late-stage models,

Overall, the transcriptomics analysis showed that cells in the hPB early-stage model is actively growing and differentiating and developing placenta structure and function that mimics the rapidly forming and maturing placenta during the first trimester, as shown by upregulating of proliferative with cell cycle and metabolism genes activated . At the late stage hPB model, genes related to ECM remodeling, hormone secretion and immune responses are highly expressed, which mimics a placenta that has evolved to have activities that support the growth and protection of the fetus and maintain the pregnancy (hormone secretion).
