# Supplementary material for: Recapitulating gestational progression: a high-throughput 3D bioprinted vascularized human placenta demonstrating stage-specific molecular and functional signatures of human pregnancy
Source: Sci Rep. 2026 Jul 28;16:24140. doi: 10.1038/s41598-026-61708-y (PMC13443107; doi:10.1038/s41598-026-61708-y)
Supplement: Supplementary file 1 [file 41598_2026_61708_MOESM2_ESM.pdf]

Supplementary Note 2

Analysis of differentially expressed proteins between gestational stage models utilizing Gene Ontology enrichment analysis and GO keywords overlap with the transcriptomics data, including epithelial barrier development, nutritional, defense and hormonal activity.

As observed in Figure 4E, there was a general overexpression of proteins related to epidermis development in early stages derived from both cell sources. Among the proteins related to metabolism that exhibited significantly different levels between two developmental stages by gene expression, protein levels identified up-regulation of those involved in glucose metabolism in the early-stage models derived from primary Tbs. Proteins related to amino acid metabolism also exhibited some differences between models, being more elevated in late-stages of the hPB model with immortalized Tbs, including KYNU which was also detected by transcriptomics analysis above (Figure 4F and 4M).

Proteins associated with defense response to xenobiotic stimulus, such as xenobiotics, or xenobiotic-metabolic enzymes, did not show significant differences in expression between the two gestational models, regardless of Tb cells used (Figure 4G and 4N). Only some drug efflux transporters, including ABCC2 and ABCG2, had elevated expression in early-stage models derived from immortalized cells. Conversely, increased levels of proteins linked to bacterial and viral responses, as well as cytokines, were markedly distinct between stages (i.e. LTF, MX1, IL-6), demonstrating a strong association with gestational age, as observed in the transcriptome study.

In general, there were no differential expression of proteins related to nutrients transport, including glucose and protein transport. Higher expression of some proteins related to amino acid transport was detected in the late-stage model, for both Tbs types, including SCL transporters such as SCL43A1 and A2. Consistent with the transcriptome results, proteins related to lipid transport exhibited the most pronounced differential changes (Figure 4H and 4O). Although there was no obvious correlation with gestational age in placenta models derived from the primary Tbs, we observed an increase in apolipoproteins (e.g APOE, APOH, APM) during the early stage of placenta development from the immortalized Tbs.

Proteins associated with the synthesis of hormones, such as estrogens and progesterone, were significantly increased in the late-stage placenta models produced from both Tbs cell types (Figure 4I and 4P). This is also supportive of placental endocrine functions during the latter stages of pregnancy when the endocrine disruptions are expected. Among those, enzymes implicated in the biosynthesis of the hormones, including HSD17B1 and CYP11A1. Similarly, levels of placental lactogen and subunits of chorionic gonadotropin were markedly upregulated in the late stage hPBs assembled with either primary or immortalized Tbs. Other proteins related to female pregnancy, such as PSGs, demonstrated markedly elevated levels in late-stage models produced from primary Tbs.

As seen for gene expression, significant changes in the levels of extracellular matrix proteins including laminins, had significantly elevated expression in the early-stage models of placenta from both TB cell types. Conversely, proteases such as MMP1 and ADAMTS9 were predominantly elevated in late-stage models (Figure 4J and 4Q).

Numerous proteins associated with angiogenesis show significant differential expression across the two gestational stage models (Figure 4K and 4R). High expression of Serpine-1, angiopoietin-like protein 4 (ANGPTL4) were detected in early-stage placental models generated from either Tbs. Conversely, proteins like WNT7A were particularly prevalent in late-stage models.
