# Supplementary material for: Recapitulating gestational progression: a high-throughput 3D bioprinted vascularized human placenta demonstrating stage-specific molecular and functional signatures of human pregnancy
Source: Sci Rep. 2026 Jul 28;16:24140. doi: 10.1038/s41598-026-61708-y (PMC13443107; doi:10.1038/s41598-026-61708-y)
Supplement: Supplementary file 2 [file 41598_2026_61708_MOESM3_ESM.pdf]

Supplementary Note 3

The metabolomics investigations of nutrient transfer activities in hPB models at early and late stages included two experimental conditions: first, we assessed whether the placenta would naturally secrete nutrients to the fetal side; in a second experimental design, metabolites were added to the apical (maternal) side, to explore enhanced transport to the basal (fetal) side. For the first experimental design (secretion from placenta), the targeted metabolomic profiling revealed that there was a trend for all three metabolites to be more abundant in the medium of the fetal side on the late-stage hPB models derived from the primary or immortalized trophoblasts, but the variations in levels (measured as area under the peak or A.U.P.). However, when a gradient was established by adding metabolites to the medium in the apical chamber, the late-stage model greatly enhanced the transfer of these nutrients, particularly glucose, and leucine. Similarly, no notable differences in levels of these metabolites were seen between stages in the hPB models produced from immortalized Tbs under the first experimental conditions. The addition of nutrients in the apical chamber led to a notable increase in glucose and leucine levels in the late-stage model, albeit to a lesser extent in the than in the model derived from primary immortalized trophoblasts cells.
